# Cationic Adsorption-Induced Microlevelling Effect: A Pathway to Dendrite-Free Zinc Anodes

**DOI:** 10.1007/s40820-025-01709-0

**Published:** 2025-03-26

**Authors:** Long Jiang, Yiqing Ding, Le Li, Yan Tang, Peng Zhou, Bingan Lu, Siyu Tian, Jiang Zhou

**Affiliations:** 1 State Key Laboratory of Oil and Gas Equipment, CNPC Tubular Goods Research Institute, Xi’an, 710077 People’s Republic of China; 2 School of Materials Science and Engineering, Hunan Provincial Key Laboratory of Electronic Packaging and Advanced Functional Materials, Central South University, Changsha, 410083 People’s Republic of China; 3 Hunan Provincial Key Defense Laboratory of High Temperature Wear-Resisting Materials and Preparation Technology, Hunan University of Science and Technology, Xiangtan, 411201 People’s Republic of China; 4 School of Physics and Electronics, Hunan University, Changsha, 410082 People’s Republic of China

**Keywords:** Aqueous zinc-ion batteries, Zinc anodes, Rare-earth cations, Microlevelling, Zinc dendrites

## Abstract

Dendrite growth represents one of the most significant challenges that impede the development of aqueous zinc-ion batteries. Herein, Gd^3+^ ions are introduced into conventional electrolytes as a microlevelling agent to achieve dendrite-free zinc electrodeposition. Simulation and experimental results demonstrate that these Gd^3+^ ions are preferentially adsorbed onto the zinc surface, which enables dendrite-free zinc anodes by activating the microlevelling effect during electrodeposition. In addition, the Gd^3+^ additives effectively inhibit side reactions and facilitate the desolvation of [Zn(H_2_O)_6_]^2+^, leading to highly reversible zinc plating/stripping. Due to these improvements, the zinc anode demonstrates a significantly prolonged cycle life of 2100 h and achieves an exceptional average Coulombic efficiency of 99.72% over 1400 cycles. More importantly, the Zn//NH_4_V_4_O_10_ full cell shows a high capacity retention rate of 85.6% after 1000 cycles. This work not only broadens the  application of metallic cations in battery electrolytes but also provides fundamental insights into their working mechanisms.

**Supplementary Information:**

The online version contains supplementary material available at 10.1007/s40820-025-01709-0.

## Highlights

- A microlevelling agent containing Gd^3+^ ions significantly enhances the reversibility and stability of zinc anodes.
- Gd^3+^ ions are preferentially adsorbed onto zinc surface, shielding zinc deposition at protrusions.
- Adsorbed Gd^3+^ ions establish a water-poor electric double layer, suppressing side reactions at the interface.

**Supplementary Information:**

The online version contains supplementary material available at 10.1007/s40820-025-01709-0.

## Graphical abstract

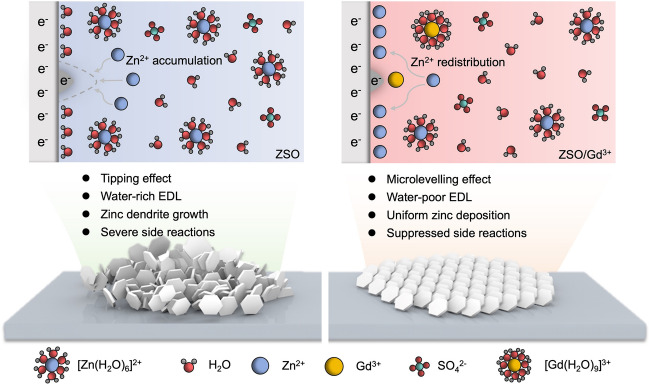

## Introduction

Aqueous zinc-ion batteries (AZIBs) are widely regarded as promising candidates for large-scale energy storage systems owing to their superior advantages in cost, safety, and  resource availability [1–3]. However, their practical employment is hindered by rapid dendrite evolution caused by uneven zinc deposition during charge/discharge [4–6]. At the microscopic scale, zinc anodes typically exhibit a rough and irregular surface morphology. This morphological irregularity results in a tipping effect where charges accumulate at protrusions on the anode surface, creating localized electric field and concentrated Zn^2+^ fluxes [7, 8].  During zinc deposition, these concentrated Zn^2+^ ions preferentially deposit on the protrusions, leading to the formation of dendrites. The growth of dendrites further exacerbates the tipping effect, resulting in uncontrollable zinc dendrite growth that can ultimately induce short circuits within the battery [9]. Additionally, water molecules tend to be adsorbed on the negatively charged zinc surface, forming a water-rich electric double layer (EDL) [10]. The interfacial water molecules, particularly those located near the dendrites, can easily trigger a series of side reactions, including zinc corrosion, hydrogen evolution reaction (HER), and by-product formation [11, 12]. Therefore, inhibiting zinc dendrite growth is essential for improving the stability and overall performance of AZIBs.

To address the issue of zinc dendrites, many strategies have been proposed, primarily focusing on anode modification [13–18], separator functionalization [19], and electrolyte engineering [20–25]. Among these strategies, incorporating functional additives into electrolytes has emerged as one of the most promising approaches. For instance, organic additives such as dimethyl sulfoxide [21] and dimethylformamide [22] have been shown to effectively inhibit zinc dendrite growth by modulating the Zn^2+^ solvation structures. However, the use of flammable organic molecules compromises battery safety and increases battery polarization. Moreover, the continuous decomposition of organic additives can lead to thickening of the interphase layer, ultimately resulting in unsatisfactory battery performance [26]. In contrast, inorganic salts containing metallic cations (e.g., Li^+^ [20], Mg^2+^ [23], Ce^3+^ [27], La^3+^ [28], Y^3+^ [29]) are highly effective and durable in regulating zinc deposition behavior. Compared to monovalent and divalent cations, trivalent cations demonstrate superior effectiveness in inhibiting dendrite growth due to their stronger electrostatic interactions with the charged zinc surface and Zn^2+^ ions. Additionally, metallic cations have been widely used as electrolyte additives in conventional zinc electroplating technologies to improve coating homogeneity and corrosion resistance [30]. Given the critical role of metallic cations in regulating the zinc–electrolyte interface, further exploit their potential and understand their working mechanisms in AZIBs is of significant importance.

**Fig. 1:**
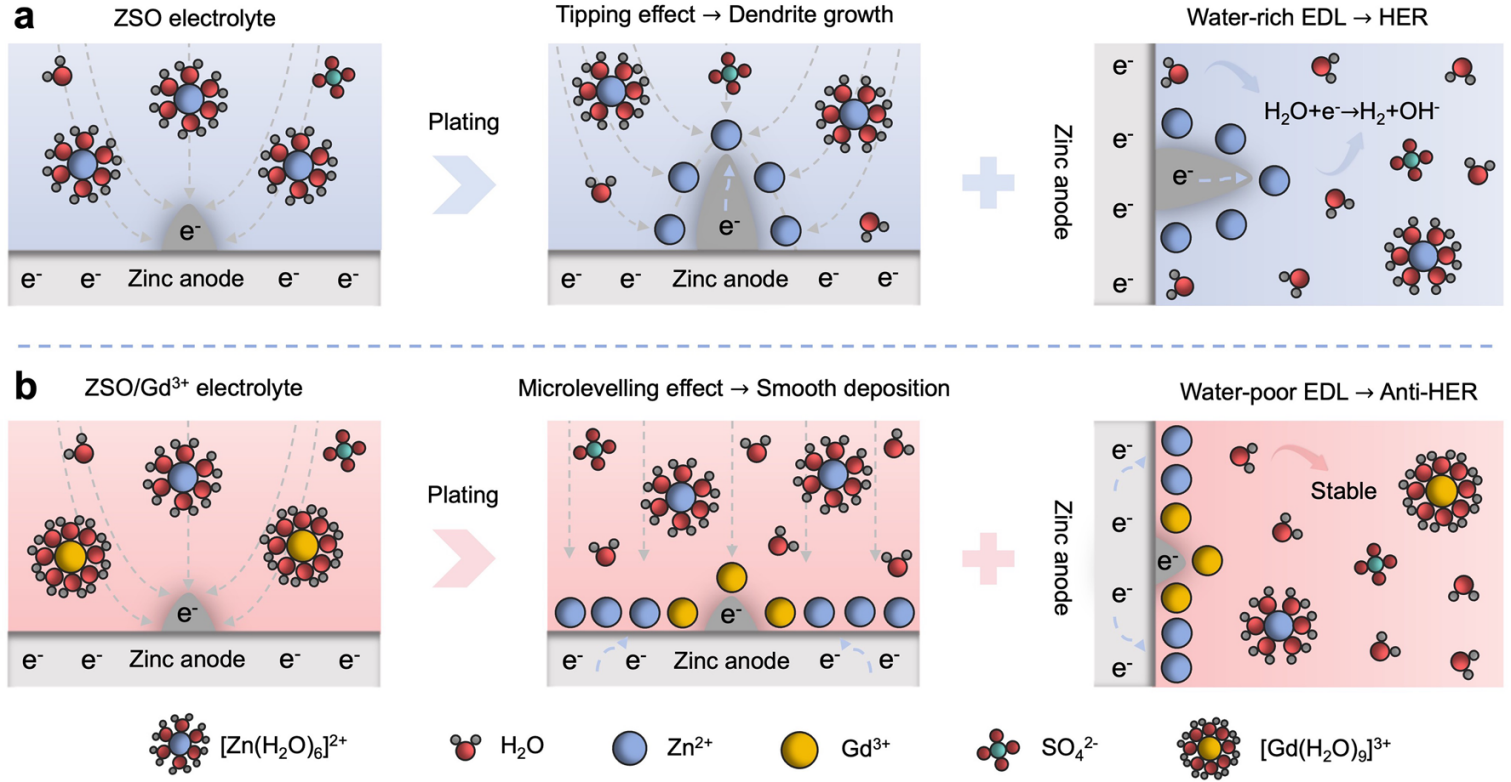
Schematic illustrations of the zinc–electrolyte interface in different electrolytes. **a** Solvated Zn^2+^ ions adsorb on protrusions due to the concentration of electric field, resulting in rampant dendrite growth in the ZSO electrolyte. Meanwhile, the water-rich EDL at the interface leads to severe HER upon zinc deposition. **b** Adsorbed Gd^3+^ ions activate the microlevelling effect by forming an electrostatic shielding layer at the interface and repels water molecules from the EDL, enabling smooth zinc deposition and suppressing HER in the ZSO/Gd^3+^ electrolyte. The gray dashed line represents the virtual profiles of the electric field

Herein, we introduce the heavy rear-earth (RE) cation Gd^3+^ into a conventional 3 M ZnSO_4_ (ZSO) electrolyte as a microlevelling agent to achieve dendrite-free zinc deposition. As schematically illustrated in Fig. 1, Gd^3+^ ions are preferentially adsorbed on to the zinc surface, thereby enhancing anode stability by activating the microlevelling effect. This mechanism stands in stark contrast to the rapid dendrite growth caused by the tipping effect in conventional ZSO electrolyte. In additional, the electrostatically adsorbed Gd^3+^ ions synergistically shield the protruding zinc surface from water molecules, suppressing detrimental side reactions such as HER and by-product formation. Consequently, highly reversible and dendrite-free zinc anodes could be achieved with the Gd^3+^ microlevelling agent. Leveraging these benefits of the Gd^3+^-containing electrolyte, the cycle life of the Zn//Zn symmetric cell significantly prolongs to over 2100 h at 1 mA cm^−2^ and 1 mAh cm^−2^. Furthermore, the Zn//Cu symmetric cell exhibits an exceptional average Coulombic efficiency (CE) of 99.72% over 1400 cycles. The Zn//NH_4_V_4_O_10_ full cell also shows a high capacity retention rate of 85.6% after 1000 cycles at 5 A g^−1^. This work provides fundamental insights into the utilization of metallic cations for developing highly efficient and reliable AZIBs.

## Experimental

### Preparation of Electrolytes

The ZSO electrolyte was prepared by dissolving 215.67 g of ZnSO_4_·7H_2_O in 250 mL of deionized (DI) water. Electrolytes with Gd^3+^ concentrations of 0.01, 0.02, 0.05, and 0.08 M were obtained by adding specific amounts of Gd_2_(SO_4_)_3_·8H_2_O powder to 30 mL of ZSO solution.

### Cell Assembling

The zinc foils with a thickness of 50 μm and a diameter of 15 mm were used as anodes. A glass fiber membrane (Whatman, GF/D) was employed as the separator. NH_4_V_4_O_10_ (NVO) powder was synthesized utilizing a hydrothermal method. Specifically, 2.106 g of NH_4_VO_3_ was dissolved in 90 mL of DI water under continuous stirring at 80 °C. Subsequently, 3.4038 g of H_2_C_2_O_4_·2H_2_O was gradually added to the mixture and kept stirring until the color changed to black green. The resulting solution was divided into three parts, and each part was kept at 140 °C for 48 h in a 50 mL polytetrafluoroethylene autoclave. After the hydrothermal process, the precipitated solids were collected and thoroughly washed with DI water. Finally, the product was dried at 60 °C for 12 h, yielding the desired NVO powder. The NVO, carbon black (Super P), and polyvinylidene fluoride were mixed in a mass ratio of 7:2:1 and dry-milled for 20 min. Subsequently, N-methyl-2-pyrrolidone was added to the solids and further milled for 10 min. The resulting slurry was uniformly coated onto stainless-steel meshes with a diameter of 12 mm. The coated meshes were placed in a vacuum oven kept at 80 °C for 12 h to obtain the NVO electrodes. The loading of NVO was maintained within the range of 0.9–1.4 mg cm^−2^.

### Electrochemical Measurements

Cyclic voltammetry (CV) measurements for Zn//Cu half cells were conducted on an electrochemical workstation (CHI660E) within the potential range of -0.2 to 1.0 V at 1 mV s^−1^. CV measurements for full cells were conducted at 0.1 mV s^−1^ within the voltage range of 0.4–1.4 V. Electrochemical impedance spectroscopy (EIS) curves of symmetric and full cells were obtained within the frequency range of 0.01–10^5^ Hz. Chronoamperometry (CA), Tafel, and linear sweep voltammetry (LSV) tests were conducted on the CHI660E electrochemical workstation. Particularly, CA curves were obtained with an overpotential of -150 mV. Tafel tests were conducted within the voltage range of -0.3 to 0.3 V at 10 mV s^−1^. LSV curves were recorded within the voltage range of -0.8 to 0.8 V at a scan rate of 5 mV s^−1^. Constant current charge–discharge and rate tests were conducted on a LAND multi-channel battery testing system (CT2001A).

### Characterizations

Structural characterization of the samples was conducted using an X-ray diffractometer (XRD, Rigaku Mini Flex 600, Cu Kα radiation, *λ* = 1.5418 Å) with a detection angle range of 5° < 2θ < 80° and a scanning speed of 10° min^−1^. The surface morphology of the zinc anodes was obtained using a scanning electron microscope (SEM, Nova Nano SEM230). The surface roughness of the zinc anodes was measured by a 3D confocal laser scanning microscope (3D-CLSM, KEYENCE VK-X1000). An in situ optical microscope (CEWEI LW750LJT) was used to visualize the interface evolution during zinc plating. An energy-dispersive X-ray spectroscopy analyzer (EDS, One Max 50) was employed to characterize the elemental distributions for zinc foils after immersion tests.

### DFT Calculations

All the periodic density functional theory (DFT) calculations were performed on Vienna Ab-initio Simulation Package (VASP). The generalized gradient approximation (GGA) implemented by Perdew–Burke–Ernzerhof (PBE) functional was adopted to describe the exchange–correlation interactions [31]. The valence electronic states were described by expanding plane wave basis set with a kinetic cutoff energy of 400 eV. The (3 × 3) cells were constructed to model the zinc (002) surface with a vacuum thickness of 15 Å. The adsorption energies (*E*_ads_) were calculated by:

1 $$E_{{{\text{ads}}}} = E_{{({\text{slab}} + {\text{Zn}})}} - E_{{{\text{slab}}}} - E_{{{\text{Zn}}}}$$

where *E*_(slab+Zn)_ and *E*_slab_ are the total energy of the model surfaces with and without adsorbed Zn^2+^ ions, respectively; *E*_Zn_ represents the total energy of an isolated Zn^2+^ ion in the gas phase. Calculations of binding energies were performed using the Gaussian09 program [32]. The PBE1PBE/SDD theoretical model was adopted for Gd^3+^ and Zn^2+^ ions, and the PBE1PBE/6-31G* theoretical model was adopted for other atoms [33]. The implicit solvation model, namely the solvation model density (SMD), was used to describe the solvation effect, and all calculations were carried out with atom pairwise dispersion correction (DFT-D3). The binding energy (*E*_b_) was calculated by:

2 $$E_{b} = E\left( {AB} \right) - E\left( A \right) - E\left( B \right)$$

where *E* represents the electronic energy of the component. The visualization of molecular structure was performed using visual molecular dynamics (VMD) [34] and Multiwfn [35].

## Results and Discussion

**Fig. 2:**
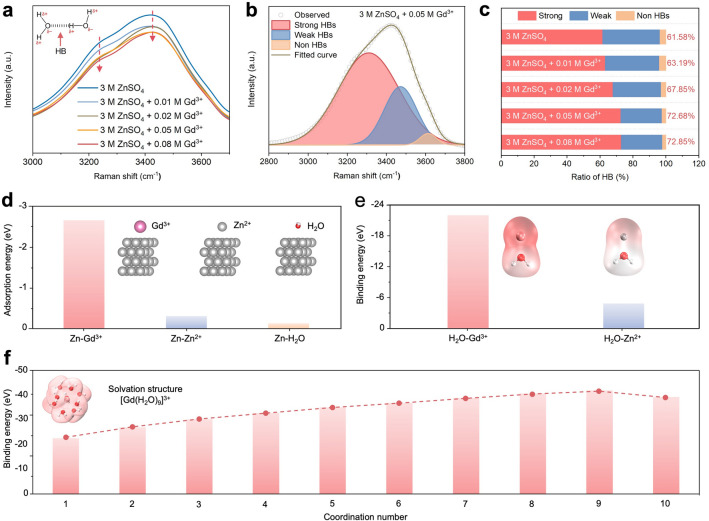
Characterizations of the electrolytes. **a** Raman spectra of the ZSO and ZSO/Gd^3+^ electrolytes with different Gd^3+^ concentrations. **b** Deconvolution of HBs and** c** corresponding ratios of different HB types in the electrolytes. **d** Absorption energies of Gd^3+^, Zn^2+^, and H_2_O on the zinc (002) surface. **e** Binding energies of Gd^3+^ and Zn^2+^ to H_2_O. **f** Calculated solvation structures of Gd^3+^ ions with H_2_O

To determine the optimal electrolyte composition, different Gd^3+^ concentrations (0.01, 0.02, 0.05, and 0.08 M) were added to the ZSO electrolyte. Figure S1 presents the Raman spectra of these electrolytes, and two characteristic peaks corresponding to the O–H and SO_4_^2−^ vibrations were observed. Notably, as the Gd^3+^ concentration increases, the intensity of the O–H vibration peak decreases (Fig. 2a), which suggests that the hydrogen bonds (HBs) among water molecules are disrupted by Gd^3+^ ions [36, 37]. Furthermore, the vibration peak is deconvoluted into three main regions representing strong, weak, and non-HBs (Fig. 2b). The ratio of strong HBs increases with higher Gd^3+^ concentrations (Fig. 2c), implying reduced water reactivity [38]. Figure S2 demonstrates the cycling stability of Zn//Zn symmetric cells. As the concentration of Gd^3+^ ions increases, the Zn//Zn symmetric cells demonstrate enhanced cycling stability. The optimal performance is achieved at a Gd^3+^ concentration of 0.05 M, which provides the best balance between enhanced stability and efficient zinc plating/stripping. Further increasing the Gd^3+^ concentration to 0.08 M results in rapid failure of the symmetric cell after approximately 300 h. This can be attributed to  significantly increased overpotentials resulting from the hindrance of zinc plating/stripping when excessive Gd^3+^ ions exist in the electrolyte. Based on these findings, the ZSO electrolyte with 0.05 M Gd^3+^ ions, denoted as ZSO/Gd^3+^ electrolyte, is selected for further investigation.

DFT calculations were performed to elucidate the working mechanisms of Gd^3+^ ions. As shown in Fig. 2d, the adsorption energy of Gd^3+^ ions on the zinc (002) surface reaches  -2.79 eV, which is significantly lower than the values for H_2_O molecules (-0.14 eV) and Zn^2+^ ions (-0.32 eV). This suggests that Gd^3+^ ions can be preferentially adsorbed on the zinc surface, playing a crucial role in activating the microlevelling effect during zinc deposition. Due to their preferential adsorption, Gd^3+^ ions will be attracted to the protrusions on the zinc surface and remain effective at their adsorption sites, causing Zn^2+^ ions to be repelled and deposited at nearby locations with relatively low heights. Consequently, smooth zinc deposition could be achieved in the ZSO/Gd^3+^ electrolyte. Meanwhile, solvated Zn^2+^ ions in the ZSO electrolyte continuously migrate from bulk electrolytes to the interface and undergo a desolvation process prior to deposition. This introduces significant amounts of free water molecules during long-term cycling, triggering severe HER on the zinc surface [39]. Figure 2e illustrates the binding energies of Zn^2+^-H_2_O and Gd^3+^-H_2_O. Compared to Zn^2+^ ions, Gd^3+^ ions exhibit a stronger binding affinity to water molecules. Moreover, the binding energy between Gd^3+^ ions and water molecules decreases until the coordination number reaches nine (Figs. 2f and S3), indicating that the most stable solvation structure for Gd^3+^ ions is [Gd(H_2_O)_9_]^3+^. This observation is consistent with the solvation number  reported in prior work and suggests that Gd^3+^ ions can efficiently disrupt the HB networks among water molecules [40, 41]. In comparison, solvated Zn^2+^ ions typically coordinate with six water molecules in the ZSO electrolyte. The strong binding affinity and high coordinating capability of Gd^3+^ ions effectively suppress water-induced interfacial side reactions , thereby contributing to the stabilization of zinc anodes in sulfate electrolytes.

**Fig. 3:**
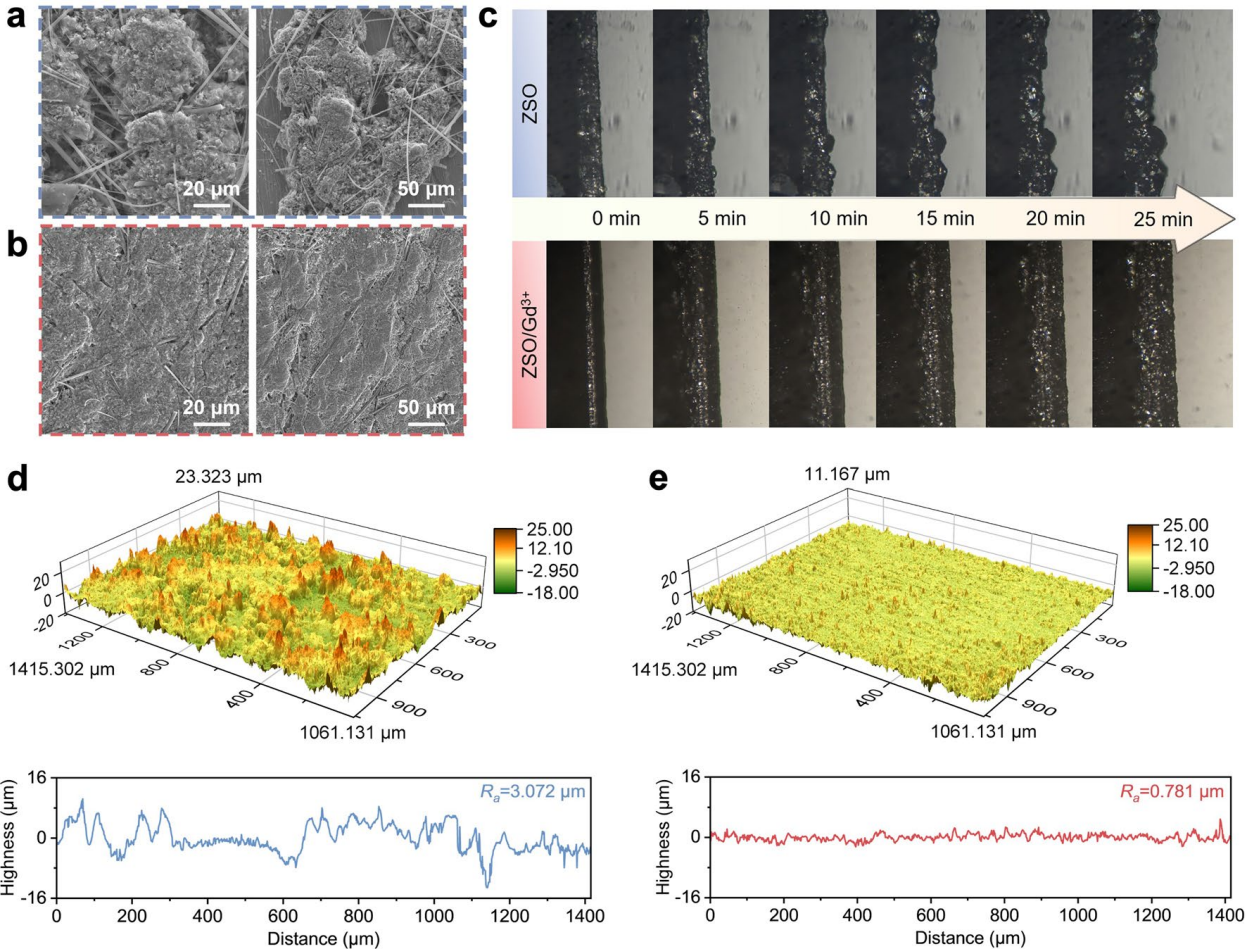
Zinc deposition behaviors in different electrolytes. SEM images of the zinc deposited at 1 mA cm^−2^ for 1 h in **a** ZSO and **b** ZSO/Gd^3+^ electrolytes. **c** In situ optical microscopy images of the zinc deposits obtained in different electrolytes. 3D-CLSM images of the zinc after cycling for 1 h in **d** ZSO and **e** ZSO/Gd^3+^ electrolytes

SEM images were obtained to investigate the morphology evolution of zinc deposits obtained in different electrolytes. In the ZSO electrolyte, zinc deposition initiates with uneven nucleation on the surface, which subsequently leads to the growth of large dendrites over time due to the tipping effect (Fig. S4). As depicted in Fig. 3a, these dendrites further develop into highly porous and moss-like clusters that can ultimately penetrate the glass fiber separator to induce battery short circuits. Notably, the formation of porous zinc deposits could be attributed to exacerbated detrimental side reactions (e.g., HER) occurring at the interface near dendrites [42]. In contrast, the zinc deposition layer obtained in the ZSO/Gd^3+^ electrolyte remains smooth and free of notable protrusions (Figs. S4 and 3b) due to the microlevelling effect induced by Gd^3+^ ions. The evenly deposited zinc ensures a homogenous distribution of electric field across the interface, thereby suppressing rampant dendrite growth and interfacial side reactions during subsequent deposition processes.

In situ optical microscopy was employed to investigate the dynamic zinc deposition behaviors in different electrolytes. Figure 3c displays optical images of the interface at different plating stages. In the ZSO electrolyte, uneven zinc deposits emerge on the surface after a plating duration of 5 min. By 25 min of plating, zinc deposits grow into large and irregular dendrites. Conversely, during zinc deposition in the ZSO/Gd^3+^ electrolyte, the interface remains uniform and the thickness of the zinc deposition layer increases smoothly. To further quantify the morphological characteristics of zinc deposits, 3D-CLSM images were obtained. As shown in Fig. 3d, the zinc deposits obtained in the ZSO electrolyte exhibit a high surface roughness (*R*_*a*_) of 3.072 μm, attributed to randomly distributed dendrites. In contrast, the *R*_*a*_ of the zinc deposits obtained in the ZSO/Gd^3+^ electrolyte significantly decreases to 0.781 μm (Fig. 3e), confirming that the microlevelling effect activated by Gd^3+^ ions effectively suppresses dendrite growth.

**Fig. 4:**
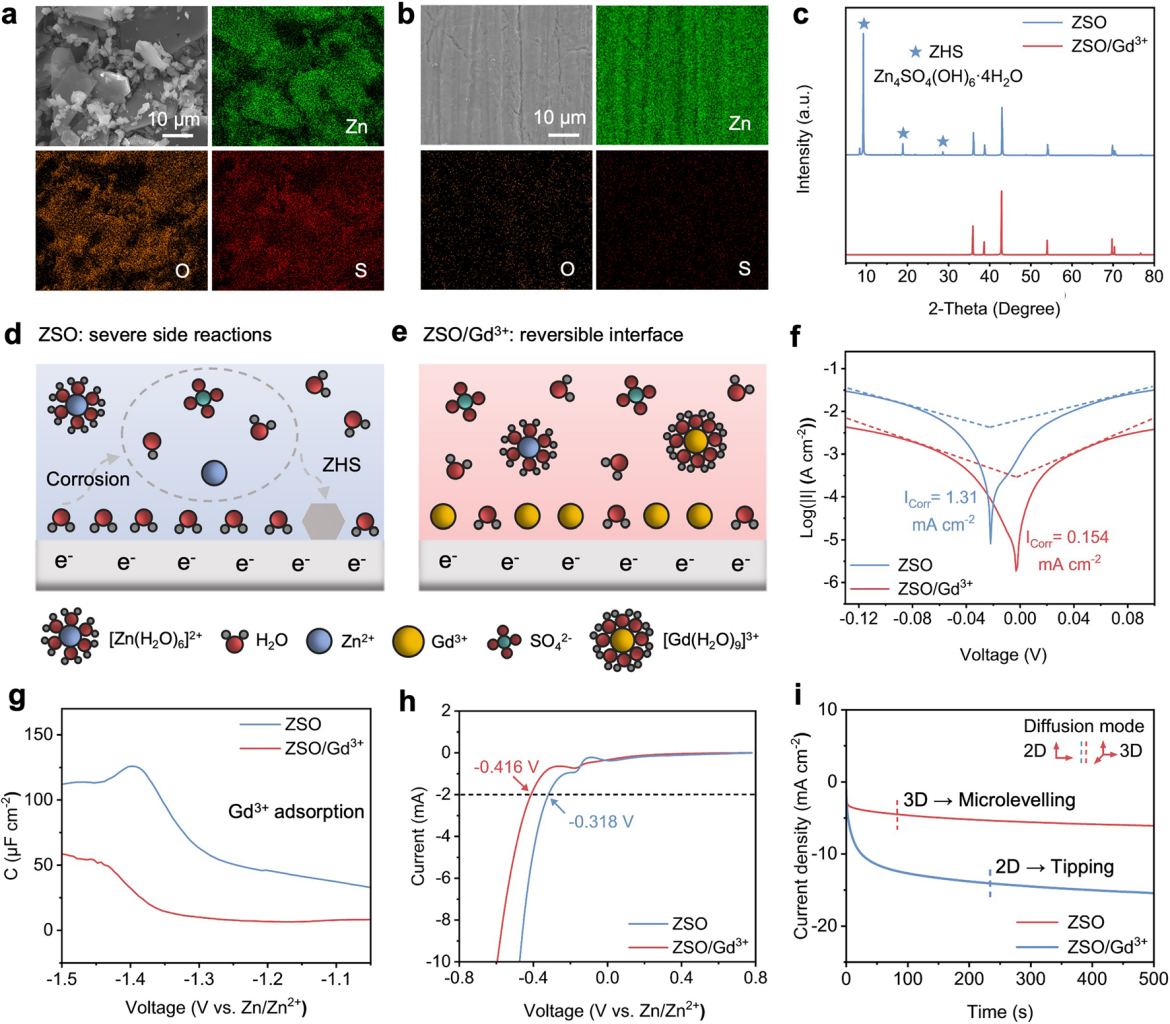
Zinc–electrolyte interface stability in different electrolytes. SEM and EDS images of the zinc surface immersed in **a** ZSO and **b** ZSO/Gd^3+^ electrolytes for 6 days. **c** XRD patterns of the zinc foil after immersion tests. Schematic illustrations of the EDL structures in **d** ZSO and **e** ZSO/Gd^3+^ electrolytes. **f** Tafel, **g** differential capacitance,** h** LSV and **i** CA curves of zinc electrodes tested in ZSO and ZSO/Gd^3+^ electrolytes

To investigate the corrosion behavior, zinc foils immersed in the ZSO and ZSO/Gd^3+^ electrolytes for 6 days were characterized using SEM. As depicted in Figs. 4a and S5, the zinc immersed in the ZSO electrolyte is covered by many by-product platelets rich in O and S, with contents reaching 59.42% and 9.28%, respectively. This observation indicates that severe zinc corrosion occurs during immersion tests in the ZSO electrolyte. When the zinc foil is immersed in the ZSO/Gd^3+^ electrolyte, the zinc surface shows no significant changes compared to its initial state (Figs. S6 and 4b). As shown in Fig. S7, only small amounts of O (5.41%) and S (0.02%) are present on the zinc surface, indicating a significant suppression of zinc corrosion in the ZSO/Gd^3+^ electrolyte. Although the pH of the ZSO/Gd^3+^ electrolyte decreases to 1.59 (Fig. S8), no significant zinc corrosion occurs in this acidic electrolyte due to the preferential adsorption of Gd^3+^ ions, consistent with the phenomenon reported in prior work [43]. The XRD results presented in Fig. 4c further corroborate these findings. For the zinc immersed in the ZSO electrolyte, large amounts of Zn_4_(SO_4_)(OH)_6_·4H_2_O (ZHS) by-products are identified. Conversely, no by-product diffraction peaks are observed for the zinc immersed in the ZSO/Gd^3+^ electrolyte.

Figure 4d, e schematically illustrates the difference between the ZSO and ZSO/Gd^3+^ electrolytes. In conventional ZSO electrolytes, spontaneous zinc corrosion occurs at the water-rich interface and results in the formation of non-ion-conducting ZHS by-products (Fig. 4d). However, the zinc anode remains stable in the ZSO/Gd^3+^ electrolyte due to the formation of a water-poor interface through the self-adsorption of Gd^3+^ ions (Fig. 4e). This Gd^3+^-mediated water-poor interface effectively inhibits the occurring of detrimental reactions (e.g., zinc corrosion, by-product formation). To further investigate zinc corrosion kinetics, Tafel tests were conducted in different electrolytes. As shown in Fig. 4f, the zinc electrode exhibits a corrosion current density of 0.154 mA cm^−2^ in the ZSO/Gd^3+^ electrolyte, which is lower than that (1.31 mA cm^−2^) of zinc obtained in the ZSO electrolyte. This indicates a substantial reduction in the zinc corrosion rate when Gd^3+^ ions are present in the electrolyte, consistent with the findings obtained from immersion tests.

Figure 4g displays the differential capacitance–voltage curves of zinc electrodes in different solutions. The ZnSO_4_ was substituted by Na_2_SO_4_ in the solution to exclude the influence of Faradaic currents during testing. Compared with the Na_2_SO_4_ solution, the zinc electrode exhibits a lower capacitance in the Gd^3+^-containing solution, which could be attributed to Gd^3+^ adsorption and its large hydrated ionic radius [44]. Furthermore, as indicated in Fig. S9, the presence of Gd^3+^ ions results in slightly reduced contact angles between the ZSO/Gd^3+^ electrolyte and various electrodes (e.g., Cu, Zn, and NVO), suggesting improved interfacial wettability. The introduction of Gd^3+^ ions also shifts the HER onset potential from -0.318 to -0.416 V at a current of 2 mA (Fig. 4h), demonstrating that the spontaneously formed Gd^3+^ adsorption layer effectively suppresses HER kinetics. Additionally, as indicated by the CA curves (Fig. 4i), the current density of the zinc anode increases significantly under a constant overpotential in the ZSO electrolyte, indicating a pronounced tipping effect induced by 2D diffusion. In contrast, the introduction of Gd^3+^ ions effectively transforms the Zn^2+^ diffusion mode to 3D, thus limiting the increase in current by suppressing zinc dendrite growth [45].

**Fig. 5:**
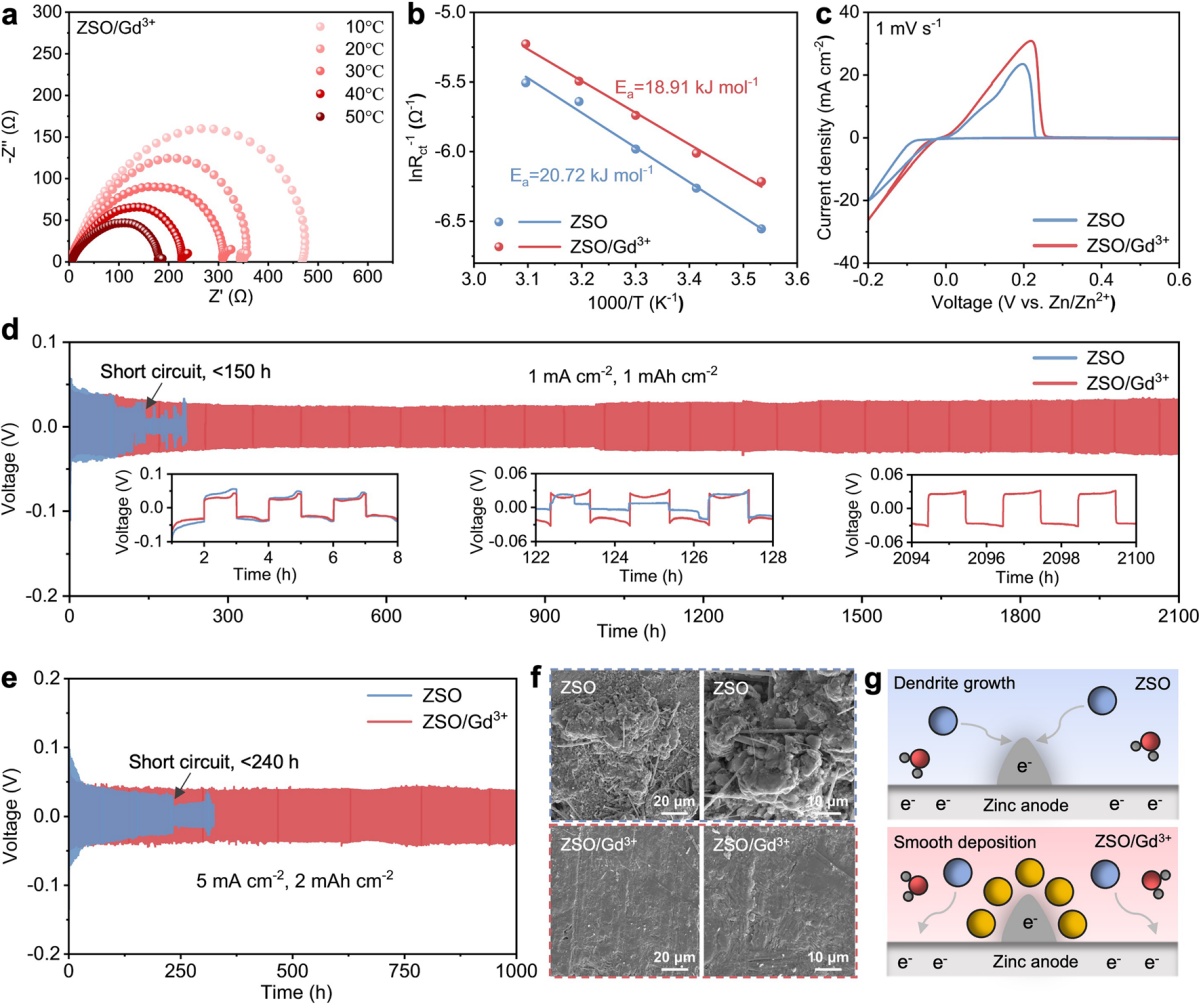
Electrochemical reversibility and stability of zinc metal anodes. **a** EIS curves of the Zn//Zn symmetric cells with the ZSO/Gd^3+^ electrolyte at different temperatures. **b** Activation energies of Zn^2+^ desolvation in different electrolytes. **c** CV curves of Zn//Cu batteries with ZSO and ZSO/Gd^3+^ electrolytes. Cycling performance of Zn//Zn symmetric cells at **d** 1 mA cm^−2^, 1 mAh cm^−2^ and **e** 5 mA cm^−2^, 2 mAh cm^−2^. **f** SEM images of the zinc anodes cycled in Zn//Zn symmetric cells. **g** Schematic illustrations of the zinc deposition behaviors in different electrolytes

To investigate zinc plating kinetics, the activation energy (*E*_a_) was calculated according to the Arrhenius equation [46]:

3 $$\frac{1}{{R_{ct} }} = A\exp \left( { - \frac{{E_{a} }}{RT}} \right)$$

where *A* is the Arrhenius constant, *R*_ct_ is the charge transfer resistance, *R* is the gas constant, and *T* is the temperature. Figures 5a and S10 present the EIS curves of the Zn//Zn symmetric cells based on ZSO/Gd^3+^ and ZSO electrolytes at different temperatures, respectively. The *E*_a_ decreases from 20.72 kJ mol^−1^ in the ZSO electrolyte to 18.91 kJ mol^−1^ in the ZSO/Gd^3+^ electrolyte (Fig. 5b), indicating that Gd^3+^ ions effectively facilitate the desolvation of Zn^2+^ ions upon zinc deposition. This reduction in desolvation energy barrier leads to a lower initial nucleation overpotential for zinc deposition in the ZSO/Gd^3+^ electrolyte (Fig. S11). Figure 5c shows the CV curves for zinc plating/stripping. In both electrolytes, the redox peaks are located at similar positions and exhibit comparable shapes, validating the stability of Gd^3+^ ions during zinc plating/stripping. In addition, Gd^3+^ ions effectively decrease the onset overpotentials for zinc plating (Fig. S12), which implies improved reversibility and kinetics of zinc deposition in the ZSO/Gd^3+^ electrolyte [47]. These improvements contribute to the superior rate capability of the Zn//Zn symmetric cell based on the ZSO/Gd^3+^ electrolyte at current densities ranging from 0.5 to 10 mA cm^−2^ (Fig. S13).

Figure 5d presents the cycling performance of Zn//Zn symmetric cells based on different electrolytes. At 1 mA cm^−2^ and 1 mAh cm^−2^, the cycling life of Zn//Zn symmetric cells is dramatically extended from approximately 150 h with the ZSO electrolyte to over 2100 h with the ZSO/Gd^3+^ electrolyte. Even at 5 mA cm^−2^ and 2 mAh cm^−2^, the Zn//Zn symmetric cell using the ZSO/Gd^3+^ electrolyte maintains a long cycle life of 1000 h with stable voltage hysteresis (Fig. 5e). In comparison, the ZSO-based Zn//Zn symmetric cell experiences dendrite-induced short circuits after 240 h. Figure 5f shows the surface morphologies of the cycled zinc anode in symmetric cells. In the ZSO electrolyte, the zinc anode exhibits a rough and highly dendritic morphology due to uneven zinc deposition. Meanwhile, the surface of the zinc anode remains smooth and free of by-product flakes when cycled in the ZSO/Gd^3+^ electrolyte, indicating the effective suppression of ZHS and Gd^3+^-containing by-products (e.g., gadolinium hydroxide and gadolinium hydroxide sulfate) that typically possess a layered structure [48, 49]. As further confirmed by the XPS spectra presented in Fig. S14, only trace amounts of Gd^3+^ and SO_4_^2−^ ions are precipitated onto the zinc surface during cycling, which is distinct from the fast and severe passivation of ZHS in conventional zinc sulfate electrolytes [49, 50]. Figure 5g schematically illustrates the significant inhibition effect of Gd^3+^ ions on dendrite growth. In the ZSO electrolyte, Zn^2+^ ions are accumulated at surface protrusions due to the tipping effect, resulting in preferential deposition of Zn^2+^ ions at these tips. However, in the ZSO/Gd^3+^ electrolyte, the protruding locations are occupied by Gd^3+^ ions, which have stronger electrostatic attraction with the negatively charged zinc surface. This creates an electrostatic shielding layer that effectively repels Zn^2+^ ions, preventing them from acquiring electrons at protrusions. Consequently, Zn^2+^ ions preferentially deposit in regions surrounding the protrusions, activating the microlevelling effect and thus promoting uniform zinc deposition.

**Fig. 6:**
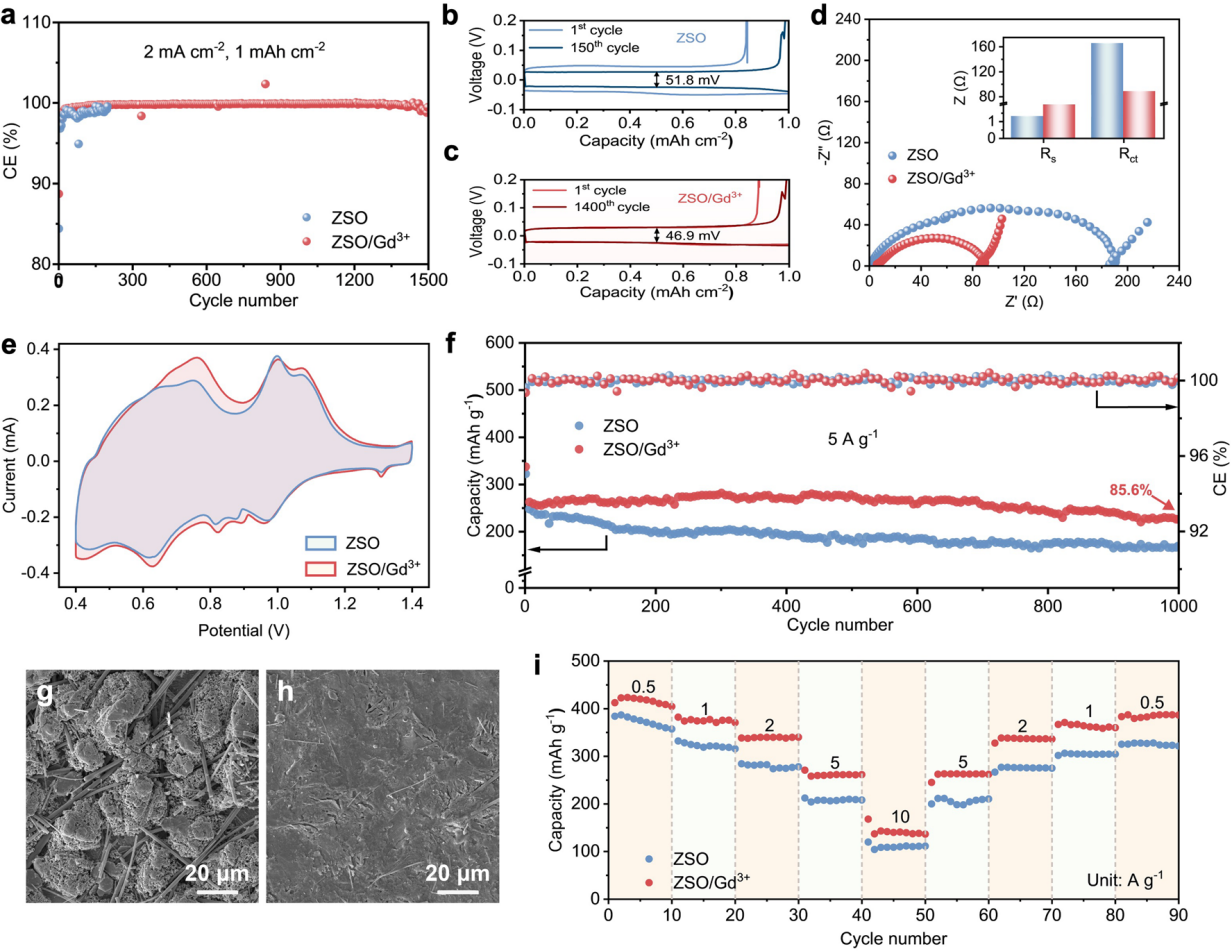
Electrochemical performance of ZSO/Gd^3+^ electrolyte. **a** CE of zinc plating/stripping in Zn//Cu batteries at 2 mA cm^−2^ and 1 mAh cm^−2^. Voltage–capacity curves of Zn//Cu batteries with **b** ZSO and **c** ZSO/Gd^3+^ electrolytes. **d** EIS and **e** CV curves of Zn//NVO full cells. **f** Cycling performance of Zn//NVO full cells at 5 A g^−1^. SEM images of the zinc anodes obtained in full cells with **g** ZSO and **h** ZSO/Gd^3+^ electrolytes after 1000 cycles at 5 A g^−1^. **i** Rate performance of Zn//NVO full cells at different current densities

To evaluate the zinc plating/stripping reversibility, Zn//Cu asymmetric cells were assembled and tested. At 2 mA cm^−2^ and 1 mAh cm^−2^, the cycle life of the Zn//Cu asymmetric cells is significantly extended from less than 200 cycles with the ZSO electrolyte to over 1400 cycles with the ZSO/Gd^3+^ electrolyte (Fig. 6a).  In addition, the ZSO-based Zn//Cu cell shows an average CE of only 98.44%, which is noticeably lower than the 99.72% achieved by the Zn//Cu cell with the ZSO/Gd^3+^ electrolyte. As shown in Fig. 6b, c, the poor zinc plating/stripping reversibility in the ZSO electrolyte results in a significant higher voltage hysteresis for the Zn//Cu cell compared to that of the ZSO/Gd^3+^ electrolyte. Such improvement in zinc plating/stripping could be mainly attributed to Gd^3+^ ions, which enable the formation of a water-poor EDL, thereby suppressing side reactions (e.g., HER, by-product formation). Similar improvements are observed for the Zn//Cu cells cycled at 1 mA cm^−2^ (Fig. S15), further confirming the effectiveness of Gd^3+^ ions in promoting zinc plating/stripping reversibility under different conditions.

To comprehensively investigate the performance of the ZSO/Gd^3+^ electrolyte in practical applications, NVO cathode material was synthesized following our previously reported method [50, 51]. The SEM image and XRD pattern shown in Fig. S16 confirm the successful synthesis of NVO powders. As shown in Fig. 6d, the Zn//NVO full cell with the ZSO electrolyte exhibits an interfacial impedance of 166 Ω, which is comparable to the value reported in prior work [51, 52]. When utilizing the ZSO/Gd^3+^ electrolyte, the interfacial impedance of the Zn//NVO full cell significantly decreases to 88.6 Ω due to enhanced charge transfer kinetics. Figure 6e presents the CV curves of the full cells at 0.1 mV s^−1^ within a voltage window of 0.4–1.4 V. No additional oxidation/reduction peaks are observed in the presence of Gd^3+^ ions, confirming their inert nature and long-term effectiveness in regulating zinc deposition. Furthermore, the full cell utilizing the ZSO/Gd^3+^ electrolyte displays higher redox peak currents compared to the ZSO-based full cell. This observation indicates that Gd^3+^ ions effectively improve zinc intercalation/deintercalation kinetics for the NVO cathode.

Figure 6f presents the cycling stability of Zn//NVO full cells at 5 A g^−1^. The ZSO-based Zn//NVO full cell exhibits fast capacity degradation due to poor zinc plating/stripping reversibility. In comparison, the Zn//NVO full cell with the ZSO/Gd^3+^ electrolyte demonstrates remarkably enhanced cycling stability with a high capacity retention rate of 85.6% after 1000 cycles, outperforming most previously reported AZIBs based on electrolytes containing other metallic cations (Table S1). Consistently, the ZSO/Gd^3+^ electrolyte effectively improves the cycling stability of the Zn//NVO full cell at 2 A g^−1^ (Fig. S17). As shown in Fig. 6g, the zinc anode cycled in the ZSO-based full cell displays a mossy and dendritic surface due to uneven zinc deposition and side reactions, leading to rapid capacity decay. However, upon introducing Gd^3+^ ions, the zinc anode in the full cell after cycling remains free of dendrites and by-products (Fig. 6h). Therefore, the long-term stability of the full cell based on the ZSO/Gd^3+^ electrolyte is effectively improved. Additionally, the ZSO/Gd^3+^ electrolyte enhances rate performance of the Zn//NVO full cells. As illustrated in Figs. 6i and S18, the specific capacities of the full cell with the ZSO/Gd^3+^ electrolyte are significantly higher than those of the ZSO-based full cell at current densities ranging from 0.5 to 10 A g^−1^, further affirming the positive impact of Gd^3+^ ions on the electrochemical performance of AZIBs.

## Conclusions

In conclusion, Gd^3+^ ions are introduced into conventional zinc sulfate electrolytes as a microlevelling agent to enable highly reversible and stable zinc anodes. The adsorbed Gd^3+^ ions not only inhibit the tipping effect at protrusions but also form a water-poor EDL near the zinc surface by substituting interfacial water molecules. Electrochemical tests and materials characterization collaboratively confirm that the adsorption of Gd^3+^ ions is beneficial in inhibiting interfacial side reactions and suppress zinc dendrite growth. Consequently, the Zn//Zn symmetric cell with the ZSO/Gd^3+^ electrolyte exhibits remarkably improved cycling stability for over 2100 h at a 1 mA cm^−2^ and 1 mAh cm^−2^. Meanwhile, when paired with the ZSO/Gd^3+^ electrolyte, the Zn//Cu asymmetric cell achieves a high average CE of 99.72% over 1400 cycles and the Zn//NVO full cell demonstrates a high capacity retention rate of 85.6% after 1000 cycles at 5 A g^−1^. This work provides novel perspectives and insights for the development of highly efficient and durable AZIBs.

## Supplementary Information

Below is the link to the electronic supplementary material.

Supplementary file1 (DOCX 13977 KB)

## References

[1] N. Pang, M. Wang, X. Wang, D. Xiong, S. Xu et al., Graphene-oxide-modified MnO_2_ composite electrode for high-performance flexible quasi-solid-state zinc-ion batteries. Mater. Sci. Eng. B **299**, 116981 (2024). 10.1016/j.mseb.2023.116981

[2] L. Song, Y. Fan, H. Fan, X. Yang, K. Yan et al., Photo-assisted rechargeable metal batteries. Nano Energy **125**, 109538 (2024). 10.1016/j.nanoen.2024.109538

[3] J. Shin, J. Lee, Y. Park, J.W. Choi, Aqueous zinc ion batteries: focus on zinc metal anodes. Chem. Sci. **11**(8), 2028–2044 (2020). 10.1039/d0sc00022a 32180925 10.1039/d0sc00022a PMC7053421

[4] S. Guo, L. Qin, T. Zhang, M. Zhou, J. Zhou et al., Fundamentals and perspectives of electrolyte additives for aqueous zinc-ion batteries. Energy Storage Mater. **34**, 545–562 (2021). 10.1016/j.ensm.2020.10.019

[5] J. Abdulla, J. Cao, D. Zhang, X. Zhang, C. Sriprachuabwong et al., Elimination of zinc dendrites by graphene oxide electrolyte additive for zinc-ion batteries. ACS Appl. Energy Mater. **4**, 4602–4609 (2021). 10.1021/acsaem.1c00224

[6] J. Yin, X. Feng, Z. Gan, Y. Gao, Y. Cheng et al., From anode to cell: synergistic protection strategies and perspectives for stabilized Zn metal in mild aqueous electrolytes. Energy Storage Mater. **54**, 623–640 (2023). 10.1016/j.ensm.2022.11.006

[7] Y. Zuo, K. Wang, P. Pei, M. Wei, X. Liu et al., Zinc dendrite growth and inhibition strategies. Mater. Today Energy **20**, 100692 (2021). 10.1016/j.mtener.2021.100692

[8] J. Zheng, Q. Zhao, T. Tang, J. Yin, C.D. Quilty et al., Reversible epitaxial electrodeposition of metals in battery anodes. Science **366**, 645–648 (2019). 10.1126/science.aax6873 31672899 10.1126/science.aax6873

[9] V. Yufit, F. Tariq, D.S. Eastwood, M. Biton, B. Wu et al., Operando visualization and multi-scale tomography studies of dendrite formation and dissolution in zinc batteries. Joule **3**(2), 485–502 (2019). 10.1016/j.joule.2018.11.002

[10] K. Qi, P. Liang, S. Wei, H. Ao, X. Ding et al., Trade-off between H_2_O-rich and H_2_O-poor electric double layers enables highly reversible Zn anodes in aqueous Zn-ion batteries. Energy Environ. Sci. **17**(7), 2566–2575 (2024). 10.1039/D4EE00147H

[11] S. Yang, H. Du, Y. Li, X. Wu, B. Xiao et al., Advances in the structure design of substrate materials for zinc anode of aqueous zinc ion batteries. Green Energy Environ. **8**(6), 1531–1552 (2023). 10.1016/j.gee.2022.08.009

[12] Y. Liang, M. Qiu, P. Sun, W. Mai, Comprehensive review of electrolyte modification strategies for stabilizing Zn metal anodes. Adv. Funct. Mater. **33**, 2304878 (2023). 10.1002/adfm.202304878

[13] Z. Xing, Y. Sun, X. Xie, Y. Tang, G. Xu et al., Zincophilic electrode interphase with appended proton reservoir ability stabilizes Zn metal anodes. Angew. Chem. Int. Ed. **62**(5), e202215324 (2023). 10.1002/anie.202215324 10.1002/anie.202215324 36446732

[14] Z. Wang, H. Chen, H. Wang, W. Huang, H. Li et al., In situ growth of a metal–organic framework-based solid electrolyte interphase for highly reversible Zn anodes. ACS Energy Lett. **7**(12), 4168–4176 (2022). 10.1021/acsenergylett.2c01958

[15] H. Du, R. Zhao, Y. Yang, Z. Liu, L. Qie et al., High-capacity and long-life zinc electrodeposition enabled by a self-healable and desolvation shield for aqueous zinc-ion batteries. Angew. Chem. Int. Ed. **61**(10), e202114789 (2022). 10.1002/anie.202114789 10.1002/anie.202114789 34939320

[16] D. Li, Y. Tang, S. Liang, B. Lu, G. Chen et al., Self-assembled multilayers direct a buffer interphase for long-life aqueous zinc-ion batteries. Energy Environ. Sci. **16**(8), 3381–3390 (2023). 10.1039/D3EE01098H

[17] Y. Zhang, Y. Zhang, J. Deng, R. Xue, S. Yang et al., In situ electrochemically-bonded self-adapting polymeric interface for durable aqueous zinc ion batteries. Adv. Funct. Mater. **34**(6), 2310995 (2024). 10.1002/adfm.202310995

[18] Z. Liu, Z. Guo, L. Fan, C. Zhao, A. Chen et al., Construct robust epitaxial growth of (101) textured zinc metal anode for long life and high capacity in mild aqueous zinc-ion batteries. Adv. Mater. **36**(5), 2305988 (2024). 10.1002/adma.202305988 10.1002/adma.202305988 37994230

[19] Y. Zhang, Z. Liu, X. Li, L. Fan, Y. Shuai et al., Loosening zinc ions from separator boosts stable Zn plating/striping behavior for aqueous zinc ion batteries. Adv. Energy Mater. **13**(42), 2302126 (2023). 10.1002/aenm.202302126

[20] Y. Yuan, S.D. Pu, M.A. Pérez-Osorio, Z. Li, S. Zhang et al., Diagnosing the electrostatic shielding mechanism for dendrite suppression in aqueous zinc batteries. Adv. Mater. **36**(9), e2307708 (2024). 10.1002/adma.202307708 37879760 10.1002/adma.202307708

[21] D. Feng, F. Cao, L. Hou, T. Li, Y. Jiao et al., Immunizing aqueous Zn batteries against dendrite formation and side reactions at various temperatures *via* electrolyte additives. Small **17**(42), e2103195 (2021). 10.1002/smll.202103195 34528386 10.1002/smll.202103195

[22] J. Cao, X. Wang, D. Zhang, R. Chanajaree, L. Zhang et al., Boosting Zn metal anode stability with a dimethylformamide additive. J. Alloys Compd. **972**, 172773 (2024). 10.1016/j.jallcom.2023.172773

[23] P. Wang, X. Xie, Z. Xing, X. Chen, G. Fang et al., Mechanistic insights of Mg^2+^-electrolyte additive for high-energy and long-life zinc-ion hybrid capacitors. Adv. Energy Mater. **11**, 2101158 (2021). 10.1002/aenm.202101158

[24] Z. Guo, Z. Liu, P. Wang, C. Zhao, X. Lu et al., Biomineralization inspired the construction of dense spherical stacks for dendrite-free zinc anodes. Nano Lett. **24**(46), 14656–14662 (2024). 10.1021/acs.nanolett.4c03749 39515822 10.1021/acs.nanolett.4c03749

[25] K. Yan, Y. Fan, F. Hu, G. Li, X. Yang et al., A “polymer-in-salt” solid electrolyte enabled by fast phase transition route for stable Zn batteries. Adv. Funct. Mater. **34**(2), 2307740 (2024). 10.1002/adfm.202307740

[26] H. Wang, A. Zhou, X. Hu, Z. Hu, F. Zhang et al., Bifunctional dynamic adaptive interphase reconfiguration for zinc deposition modulation and side reaction suppression in aqueous zinc ion batteries. ACS Nano **17**(12), 11946–11956 (2023). 10.1021/acsnano.3c04155 37318040 10.1021/acsnano.3c04155

[27] Z. Hu, F. Zhang, Y. Zhao, H. Wang, Y. Huang et al., A self-regulated electrostatic shielding layer toward dendrite-free Zn batteries. Adv. Mater. **34**(37), e2203104 (2022). 10.1002/adma.202203104 35765154 10.1002/adma.202203104

[28] R. Zhao, H. Wang, H. Du, Y. Yang, Z. Gao et al., Lanthanum nitrate as aqueous electrolyte additive for favourable zinc metal electrodeposition. Nat. Commun. **13**(1), 3252 (2022). 10.1038/s41467-022-30939-8 35668132 10.1038/s41467-022-30939-8 PMC9170708

[29] Y. Chen, S. Zhou, J. Li, J. Kang, S. Lin et al., Non-expendable regulator enables durable and deep cycling aqueous zinc batteries. Adv. Energy Mater. **14**(25), 2400398 (2024). 10.1002/aenm.202400398

[30] M. Forsyth, B. Hinton, Rare Earth-based Corrosion Inhibitors (Woodhead Publishing, Cambridge, 2014)

[31] J.P. Perdew, K. Burke, M. Ernzerhof, Generalized gradient approximation made simple. Phys. Rev. Lett. **77**(18), 3865–3868 (1996). 10.1103/physrevlett.77.3865 10062328 10.1103/PhysRevLett.77.3865

[32] Y. Liu, S. Liu, X. Xie, A functionalized separator enables dendrite-free Zn anode *via* metal-polydopamine coordination chemistry. InfoMat **5**(3), e12374 (2023). 10.1002/inf2.12374

[33] W. Xin, Y. Cui, Y. Qian, T. Liu, X.-Y. Kong et al., High-efficiency dysprosium-ion extraction enabled by a biomimetic nanofluidic channel. Nat. Commun. **15**(1), 5876 (2024). 10.1038/s41467-024-50237-9 38997277 10.1038/s41467-024-50237-9 PMC11245470

[34] W. Humphrey, A. Dalke, K. Schulten, VMD: visual molecular dynamics. J. Mol. Graph. **14**(1), 33–38 (1996). 10.1016/0263-7855(96)00018-5 8744570 10.1016/0263-7855(96)00018-5

[35] T. Lu, F. Chen, Multiwfn: a multifunctional wavefunction analyzer. J. Comput. Chem. **33**, 580–592 (2012). 10.1002/jcc.22885 22162017 10.1002/jcc.22885

[36] F. Ming, Y. Zhu, G. Huang, A.-H. Emwas, H. Liang et al., Co-solvent electrolyte engineering for stable anode-free zinc metal batteries. J. Am. Chem. Soc. **144**(16), 7160–7170 (2022). 10.1021/jacs.1c12764 35436108 10.1021/jacs.1c12764

[37] Y. Wang, Z. Wang, W.K. Pang, W. Lie, J.A. Yuwono et al., Solvent control of water O-H bonds for highly reversible zinc ion batteries. Nat. Commun. **14**(1), 2720 (2023). 10.1038/s41467-023-38384-x 37169771 10.1038/s41467-023-38384-x PMC10175258

[38] S. Chen, P. Sun, J. Humphreys, P. Zou, M. Zhang et al., *N*, *N*-dimethylacetamide-diluted nitrate electrolyte for aqueous Zn//LiMn_2_O_4_ hybrid ion batteries. ACS Appl. Mater. Interfaces **13**(39), 46634–46643 (2021). 10.1021/acsami.1c12911 34570470 10.1021/acsami.1c12911

[39] W. Ma, S. Wang, X. Wu, W. Liu, F. Yang et al., Tailoring desolvation strategies for aqueous zinc-ion batteries. Energy Environ. Sci. **17**(14), 4819–4846 (2024). 10.1039/d4ee00313f

[40] C. Clavaguéra, F. Calvo, J.-P. Dognon, Theoretical study of the hydrated Gd^3+^ ion: structure, dynamics, and charge transfer. J. Chem. Phys. **124**(7), 074505 (2006). 10.1063/1.2167647 10.1063/1.2167647 16497055

[41] T. Sun, Q. Nian, X. Ren, Z. Tao, Hydrogen-bond chemistry in rechargeable batteries. Joule **7**(12), 2700–2731 (2023). 10.1016/j.joule.2023.10.010

[42] M. Zhou, S. Guo, J. Li, X. Luo, Z. Liu et al., Surface-preferred crystal plane for a stable and reversible zinc anode. Adv. Mater. **33**, e2100187 (2021). 10.1002/adma.202100187 33864653 10.1002/adma.202100187

[43] Q. Nian, X. Luo, D. Ruan, Y. Li, B.-Q. Xiong et al., Highly reversible zinc metal anode enabled by strong Brønsted acid and hydrophobic interfacial chemistry. Nat. Commun. **15**(1), 4303 (2024). 10.1038/s41467-024-48444-5 38773073 10.1038/s41467-024-48444-5 PMC11109197

[44] E.R. Nightingale Jr., Phenomenological theory of ion solvation: effective radii of hydrated ions. J. Phys. Chem. **63**(9), 1381–1387 (1959). 10.1021/j150579a011

[45] W. Zhang, Y. Dai, R. Chen, Z. Xu, J. Li et al., Highly reversible zinc metal anode in a dilute aqueous electrolyte enabled by a pH buffer additive. Angew. Chem. Int. Ed. **62**(5), e202212695 (2023). 10.1002/anie.202212695 10.1002/anie.202212695 PMC10107295 36375075

[46] Q. Zou, Z. Liang, W. Wang, D. Dong, Y.-C. Lu, A nuclei-rich strategy for highly reversible dendrite-free zinc metal anodes. Energy Environ. Sci. **16**(12), 6026–6034 (2023). 10.1039/d3ee03246a

[47] Y. Zhu, J. Yin, X. Zheng, A.-H. Emwas, Y. Lei et al., Concentrated dual-cation electrolyte strategy for aqueous zinc-ion batteries. Energy Environ. Sci. **14**(8), 4463–4473 (2021). 10.1039/d1ee01472b

[48] K.-H. Lee, S.-H. Byeon, Extended members of the layered rare-earth hydroxide family, RE_2_(OH)_5_NO_3_·nH_2_O (RE = Sm, Eu, and Gd): synthesis and anion-exchange behavior. Eur. J. Inorg. Chem. **2009**(7), 929–936 (2009). 10.1002/ejic.200801052

[49] B. Wang, C. Gong, J. Wang, X. Wang, J.-G. Li, Oxysulfate, oxysulfide, and oxide red-phosphors from one single hydroxyl sulfate precursor (Gd,Eu)_2_(OH)_4_SO_4_⋅nH_2_O: phase evolution, and photoluminescence. Adv. Powder Technol. **34**(12), 104271 (2023). 10.1016/j.apt.2023.104271

[50] W. Lim, X. Li, D. Reed. Understanding the role of zinc hydroxide sulfate and its analogues in mildly acidic aqueous zinc batteries: A review. Small Methods. **8**, 2300965 (2024). 10.1002/smtd.202300965 10.1002/smtd.202300965 37803913

[51] Y. Ding, X. Zhang, T. Wang, B. Lu, Z. Zeng et al., A dynamic electrostatic shielding layer toward highly reversible Zn metal anode. Energy Storage Mater. **62**, 102949 (2023). 10.1016/j.ensm.2023.102949

[52] S. Yao, Y. Sun, L. Pan, Pre-removing partial ammonium ion induces vanadium vacancy assist NH_4_V_4_O_10_ as a high-performance aqueous zinc ion battery cathode. Appl. Surf. Sci. **672**, 160785 (2024). 10.1016/j.apsusc.2024.160785

