# Supplementary material for: Cationic Adsorption-Induced Microlevelling Effect: A Pathway to Dendrite-Free Zinc Anodes
Source: Nanomicro Lett. 2025 Mar 26;17:202. doi: 10.1007/s40820-025-01709-0 (PMC11947342; doi:10.1007/s40820-025-01709-0)
Supplement: Supplementary file 1 — Supplementary file1 (DOCX 13977 KB) [file 40820_2025_1709_MOESM1_ESM.docx]

# Supporting Information for

**Cationic Adsorption-Induced Microlevelling Effect: A Pathway to Dendrite-Free Zinc Anodes**

Long Jiang^1,#,^*, Yiqing Ding^2,#^, Le Li^1^, Yan Tang^2^, Peng Zhou^3^, Bingan Lu^4^, and Siyu Tian^2,^*, and Jiang Zhou^2,^*

^1^ State Key Laboratory of Oil and Gas Equipment, CNPC Tubular Goods Research Institute, Xi’an, Shaanxi 710077, P. R. China

^2^ School of Materials Science and Engineering, Hunan Provincial Key Laboratory of Electronic Packaging and Advanced Functional Materials, Central South University, Changsha, Hunan 410083, P. R. China

^3^ Hunan Provincial Key Defense Laboratory of High Temperature Wear-Resisting Materials and Preparation Technology, Hunan University of Science and Technology, Xiangtan, Hunan 411201, P. R. China

^4^ School of Physics and Electronics, Hunan University, Changsha, Hunan 410082, P. R. China

^#^Long Jiang and Yiqing Ding contributed equally to this work.

*Corresponding authors. E-mail: [jianglong003@cnpc.com.cn](mailto:jianglong003@cnpc.com.cn) (Long Jiang); [siyu_tian@csu.edu.cn](mailto:siyu_tian@csu.edu.cn) (Siyu Tian); [zhou_jiang@csu.edu.cn](mailto:zhou_jiang@csu.edu.cn) (Jiang Zhou)

## Supplementary Figures and Tables


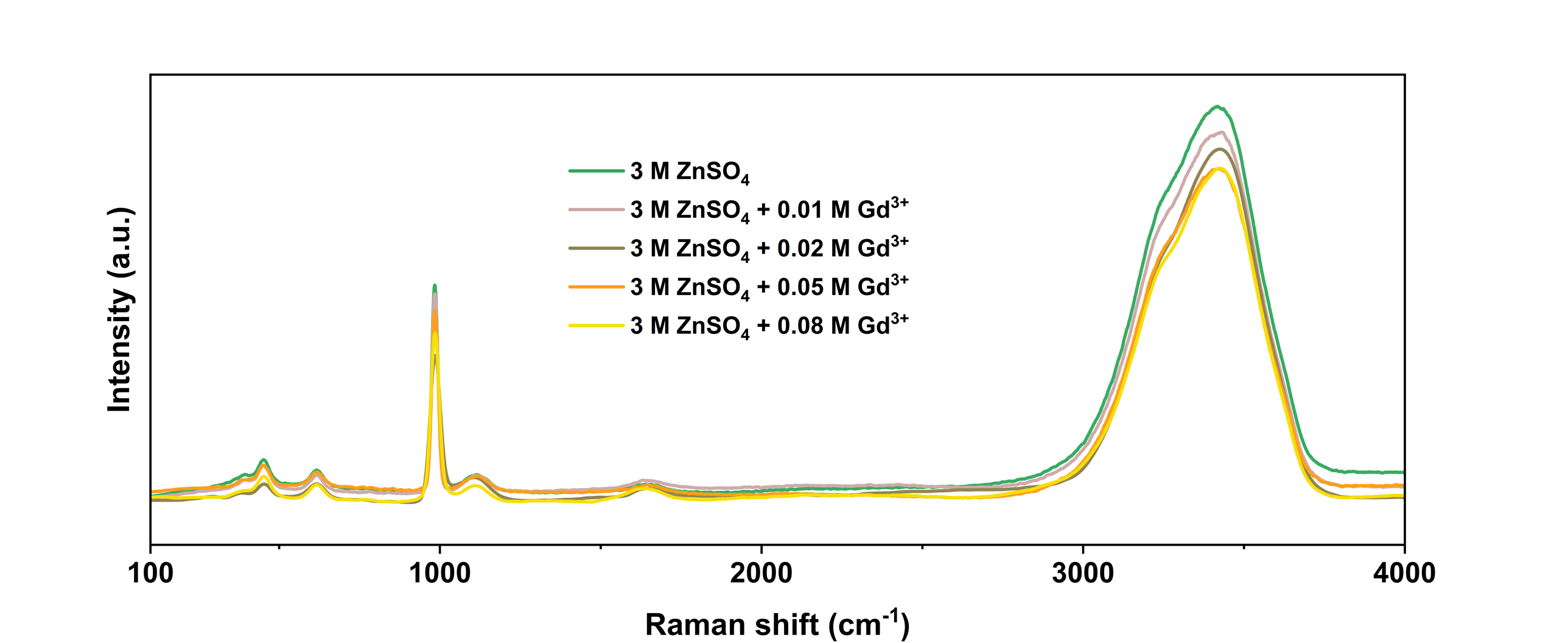


**Fig. S1** Raman spectra of ZSO and ZSO/Gd^3+^ electrolytes with different Gd^3+^ concentrations.


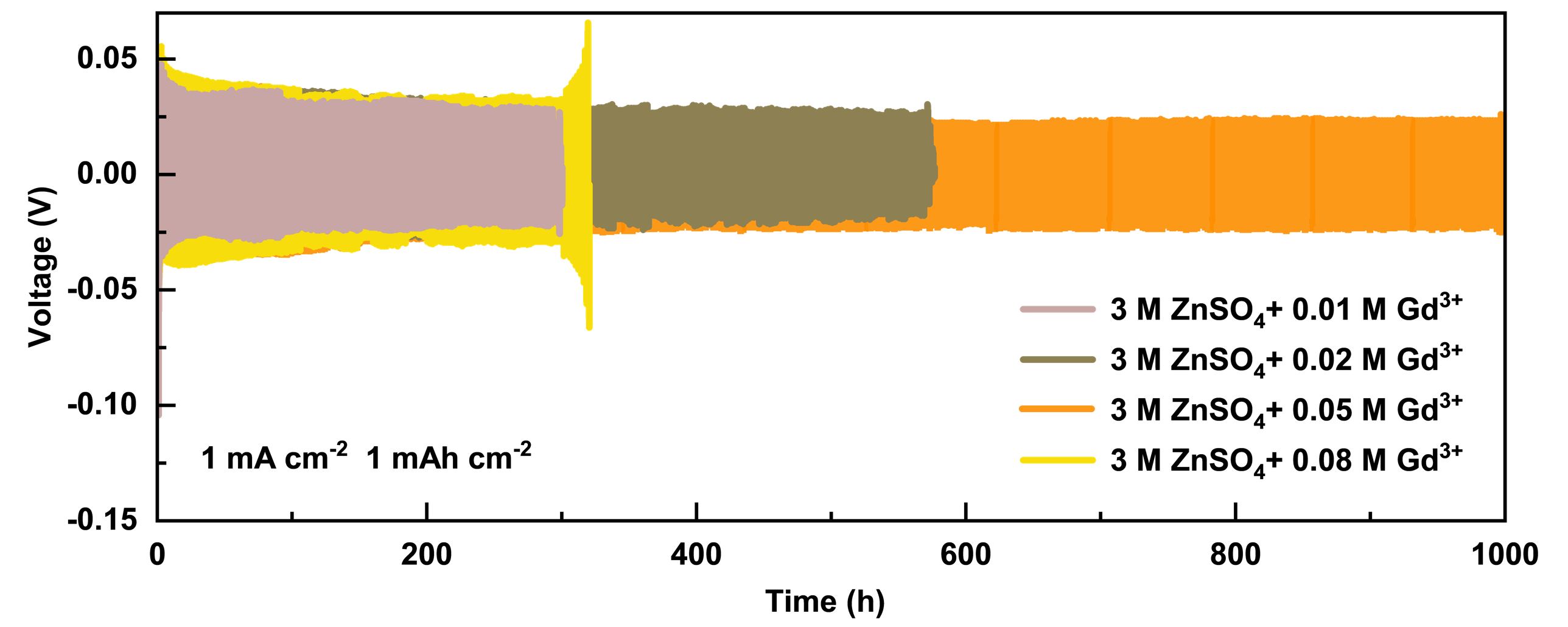


**Fig. S2** Cycling performance of the Zn//Zn symmetric cells with the electrolytes containing different Gd^3+^ concentrations at 1 mA cm^-2^ and 1 mAh cm^-2^


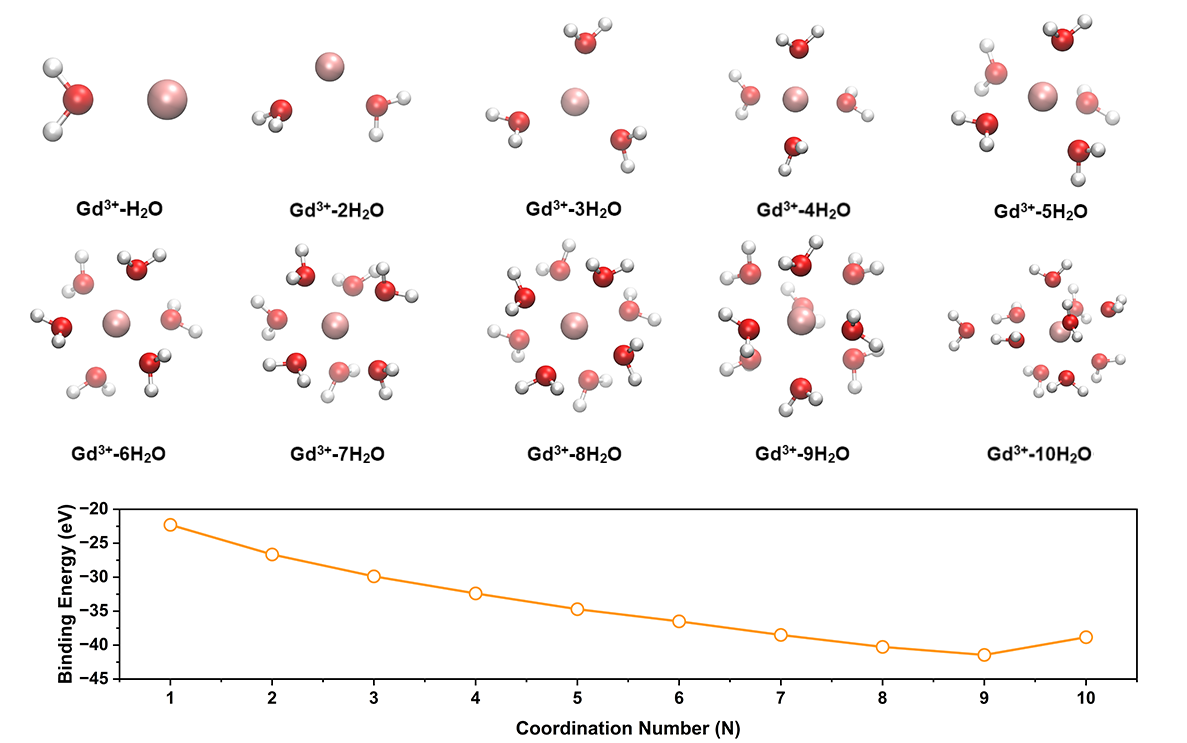


**Fig. S3** Calculations of Gd^3+^-H_2_O coordination number. The binding energy reaches the lowest at a coordination number of 9, which suggests [Gd(H_2_O)_9_]^3+^ is the most stable solvation structure for Gd^3+^ ions


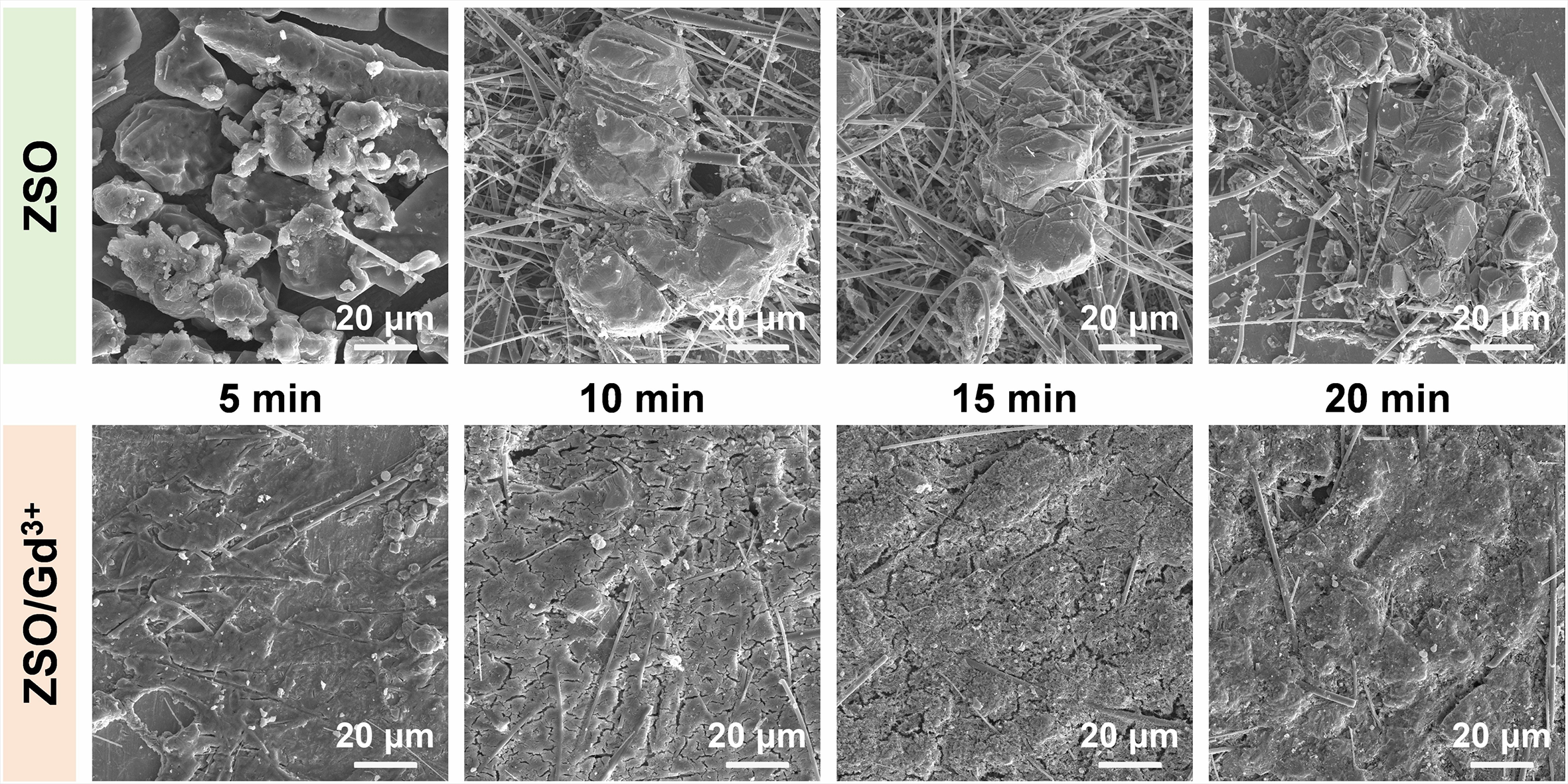


**Fig. S4** Morphology evolution of the zinc deposits over time in ZSO and ZSO/Gd^3+^ electrolytes under a current density of 1 mA cm^-2^


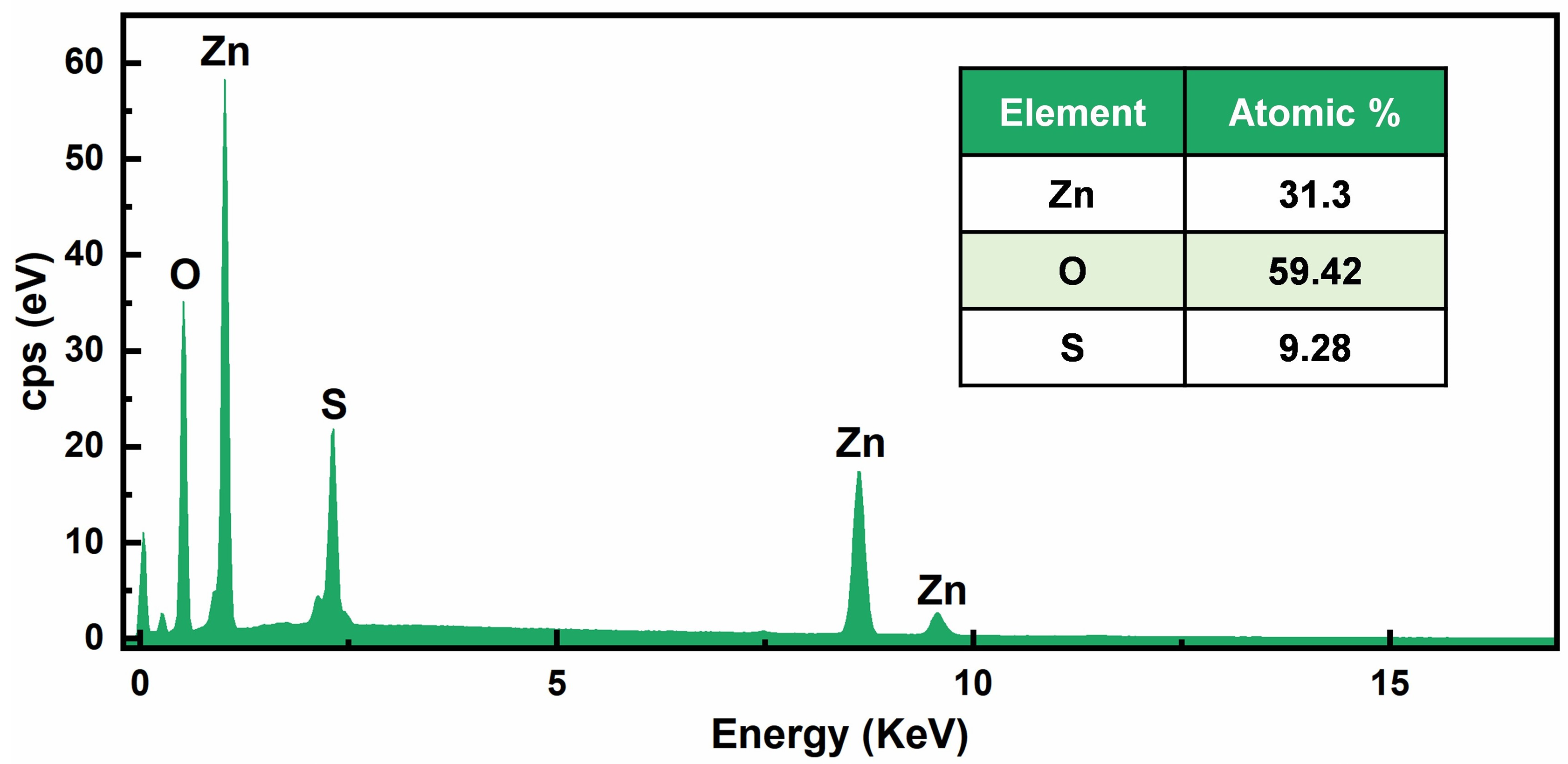


**Fig. S5** Elemental distribution of the zinc surface soaked in the ZSO electrolyte for 6 days


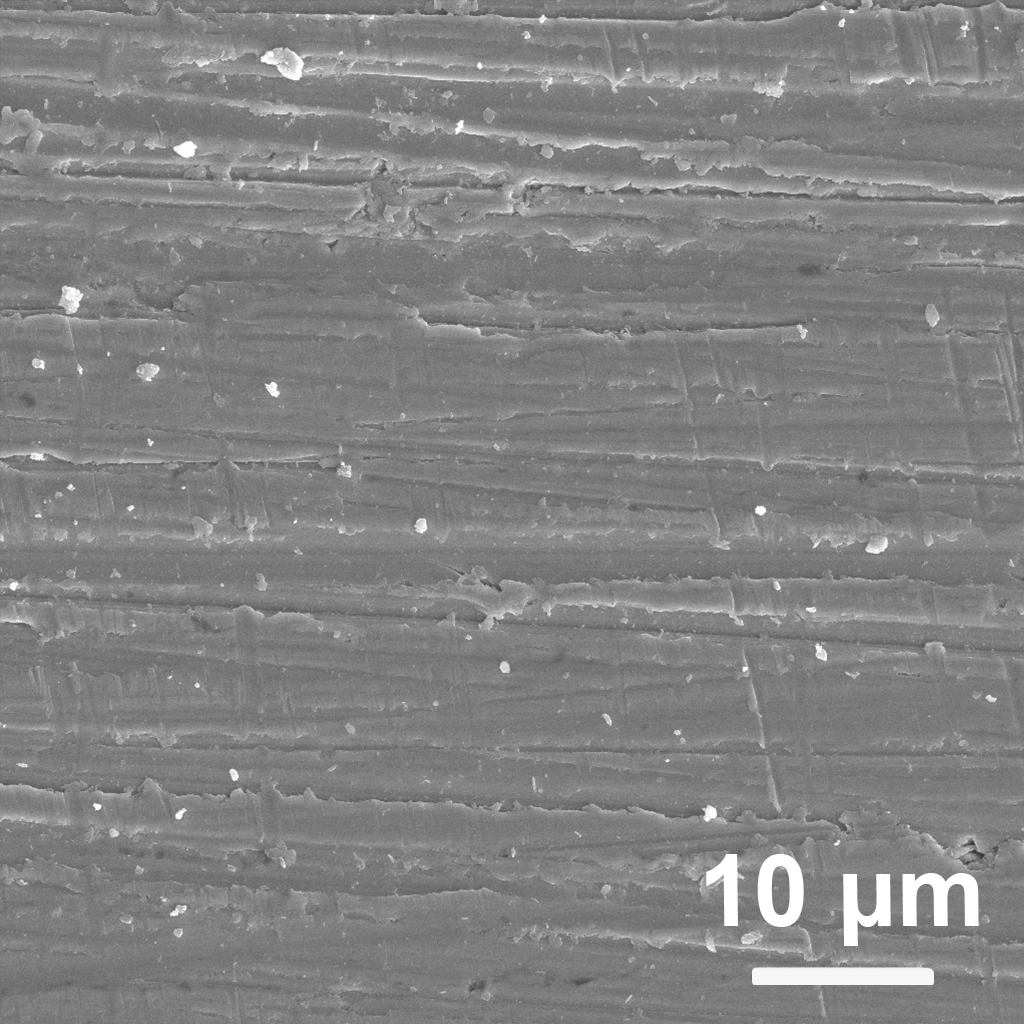


**Fig. S6** SEM image of the initial zinc surface


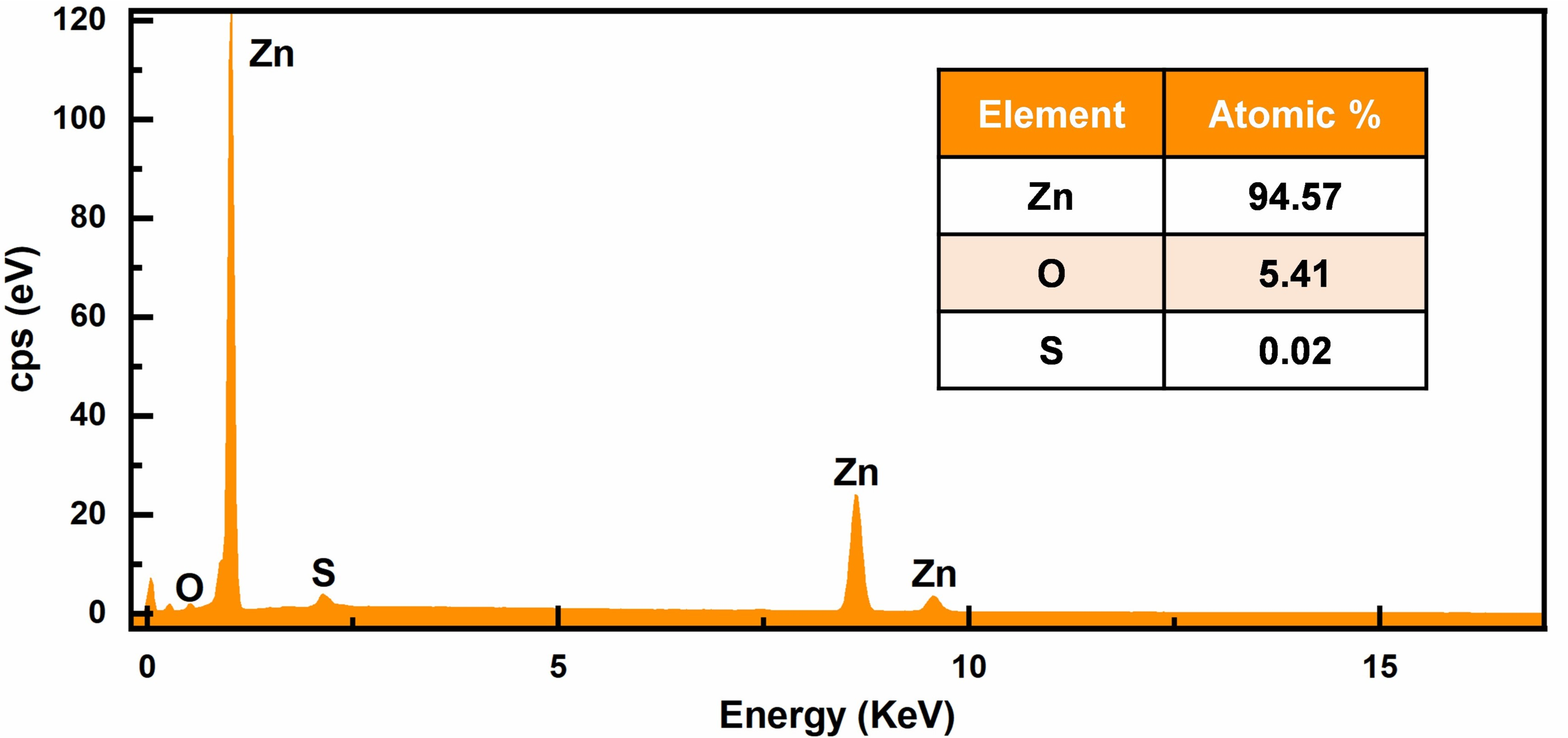


**Fig. S7** Elemental distribution of the zinc surface soaked in the ZSO/Gd^3+^ electrolyte for 6 days


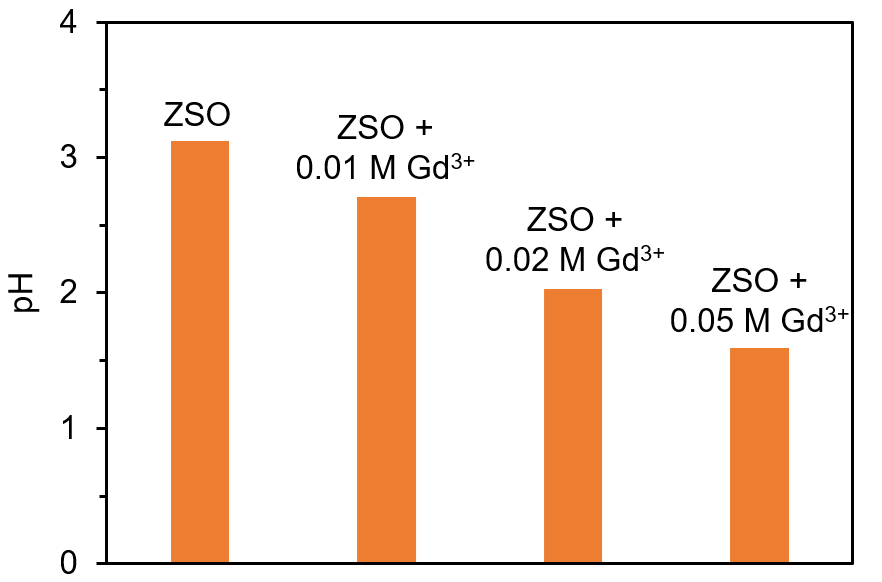


**Fig. S8** pH values of the ZSO electrolytes containing various Gd^3+^ concentrations


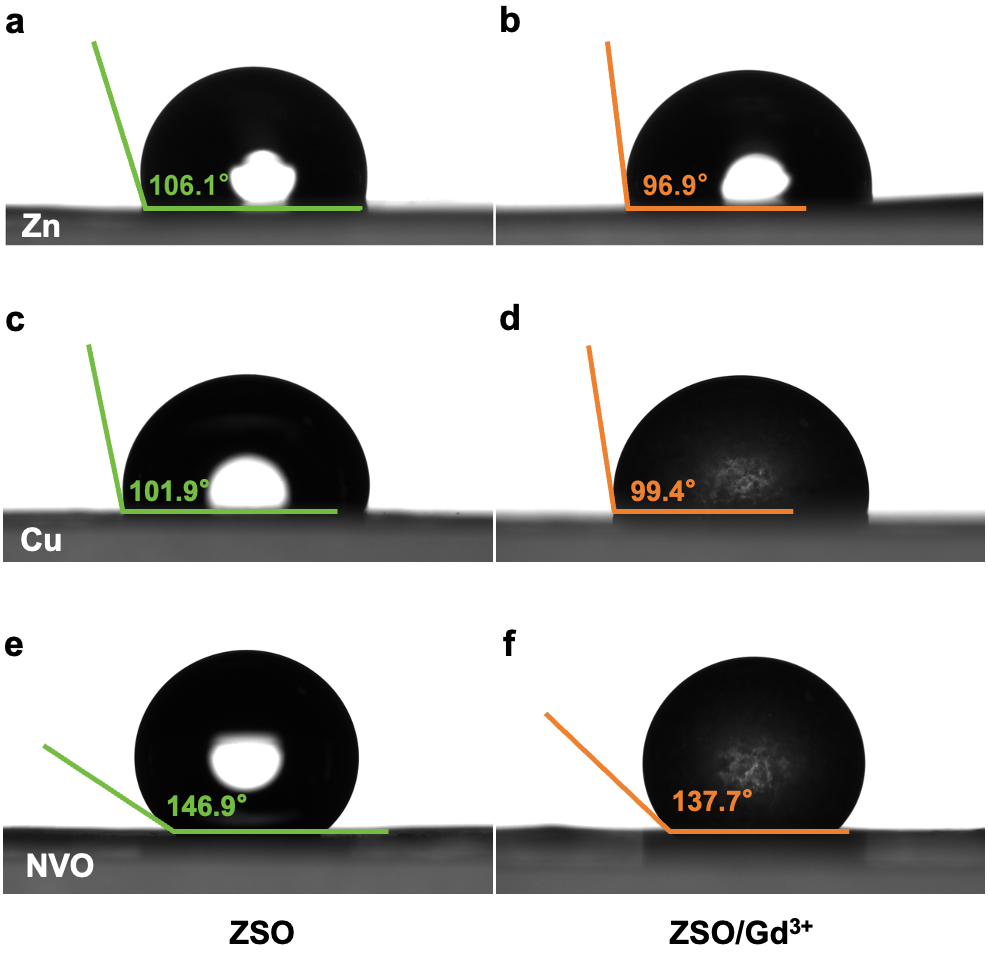


**Fig. S9** Contact angles between **a** ZSO and Zn, **b** ZSO/Gd^3+^ and Zn; **c** ZSO and Cu, d ZSO/Gd^3+^ and Cu; **e** ZSO and NVO, **f** ZSO/Gd^3+^ and NVO


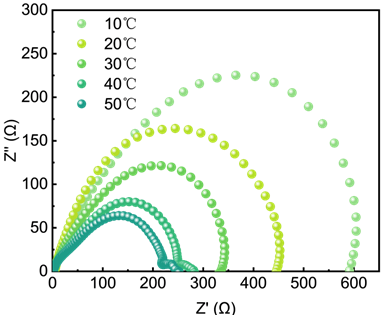


**Fig. S10** EIS curves of Zn//Zn symmetric cells using ZSO electrolyte at temperatures ranging from 10 to 50 °C


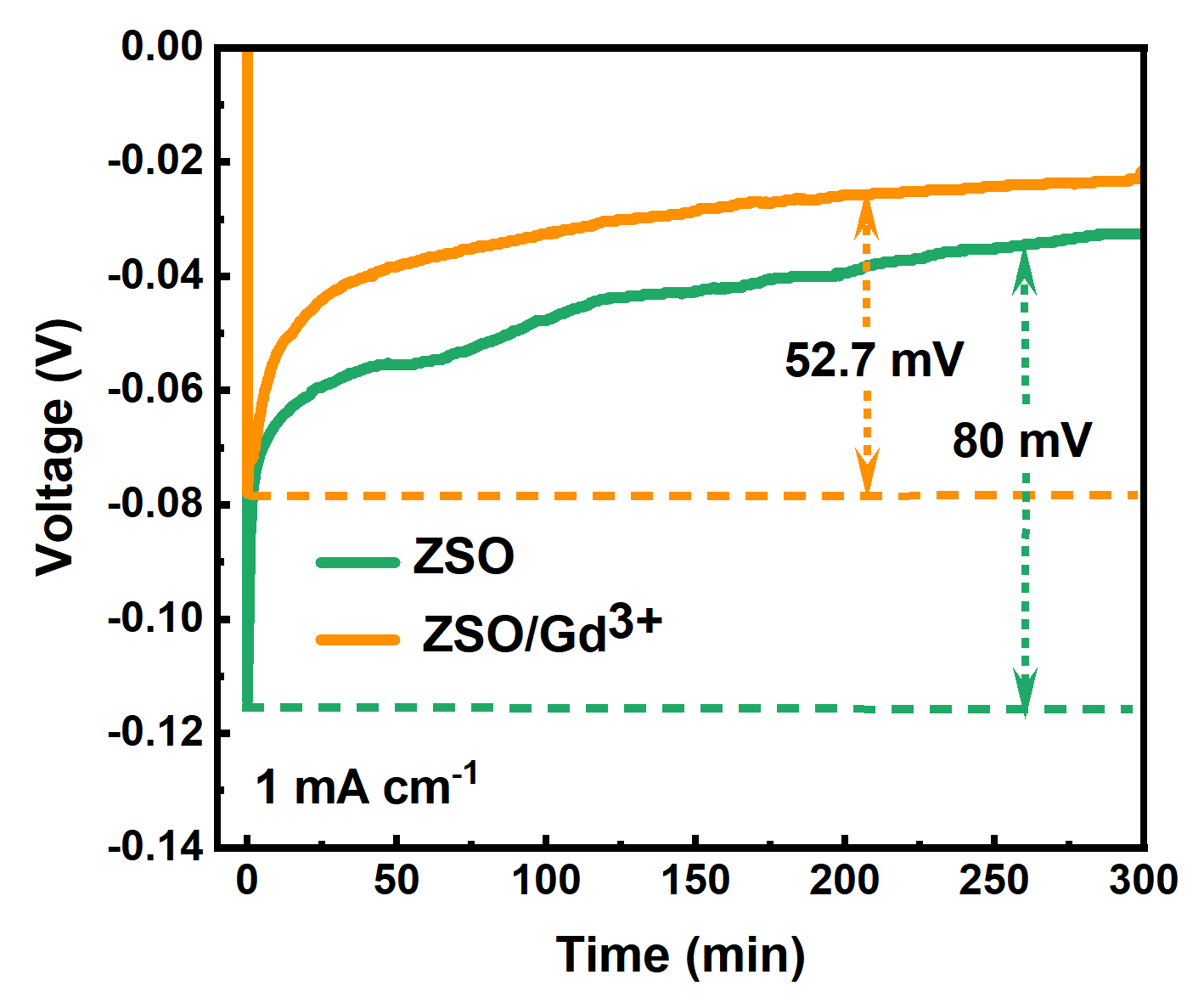


**Fig. S11** Nucleation overpotentials at 1 mA cm^-2^


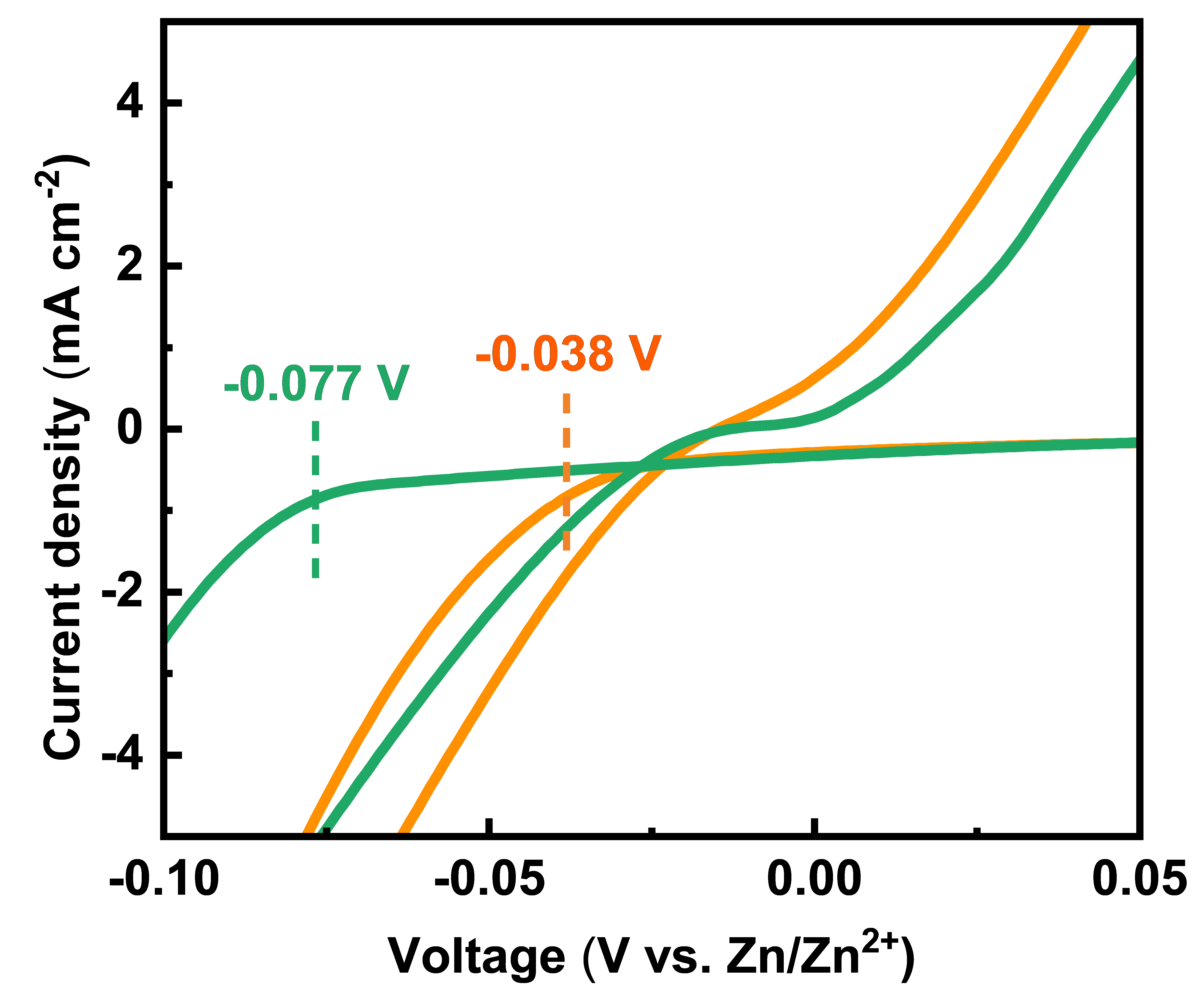


**Fig. S12** CV curves of Zn//Cu batteries with ZSO and ZSO/Gd^3+^ electrolytes showing the zinc plating process


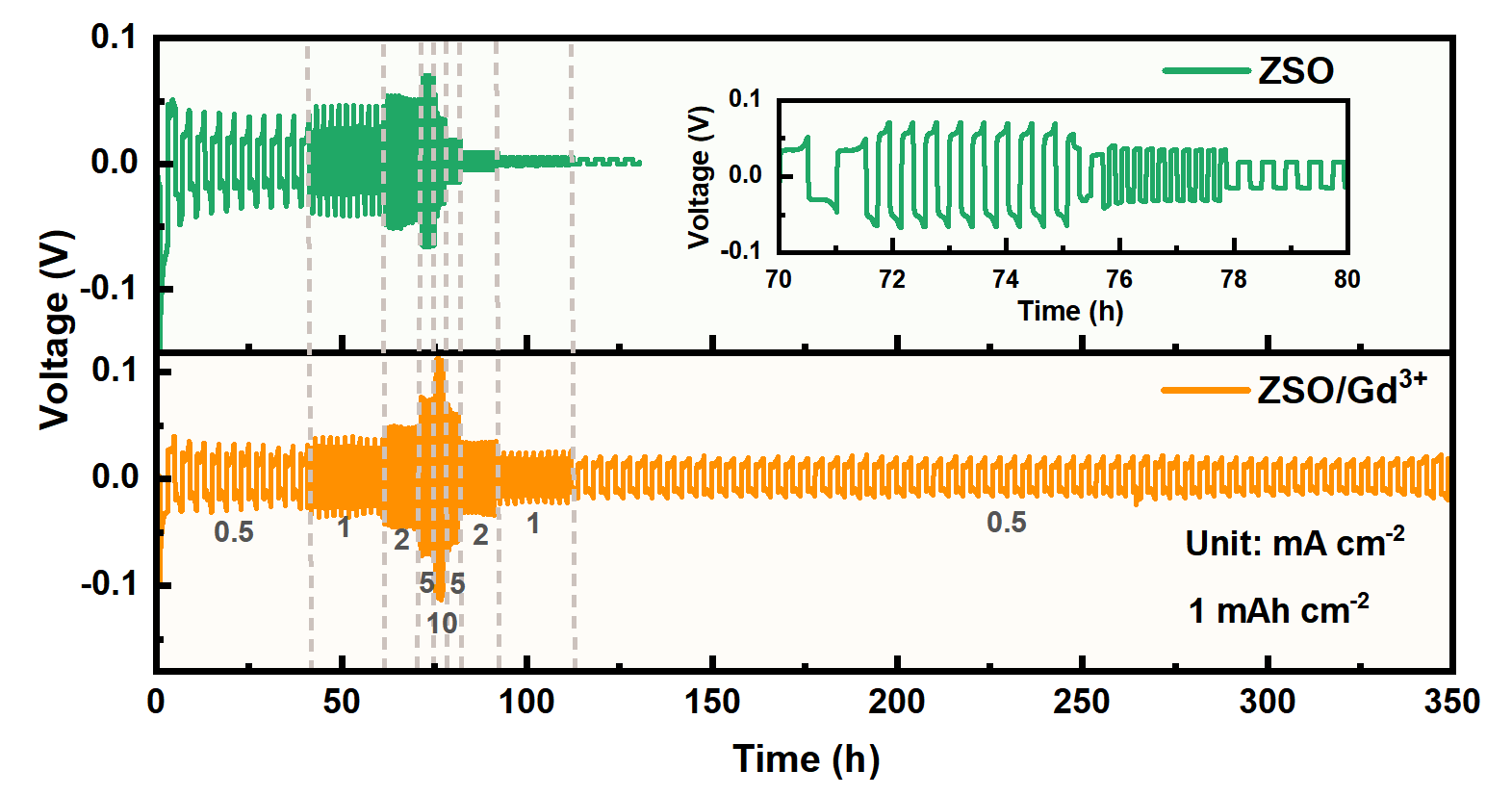


**Fig. S13** Rate capability of Zn//Zn symmetric cells with different electrolytes


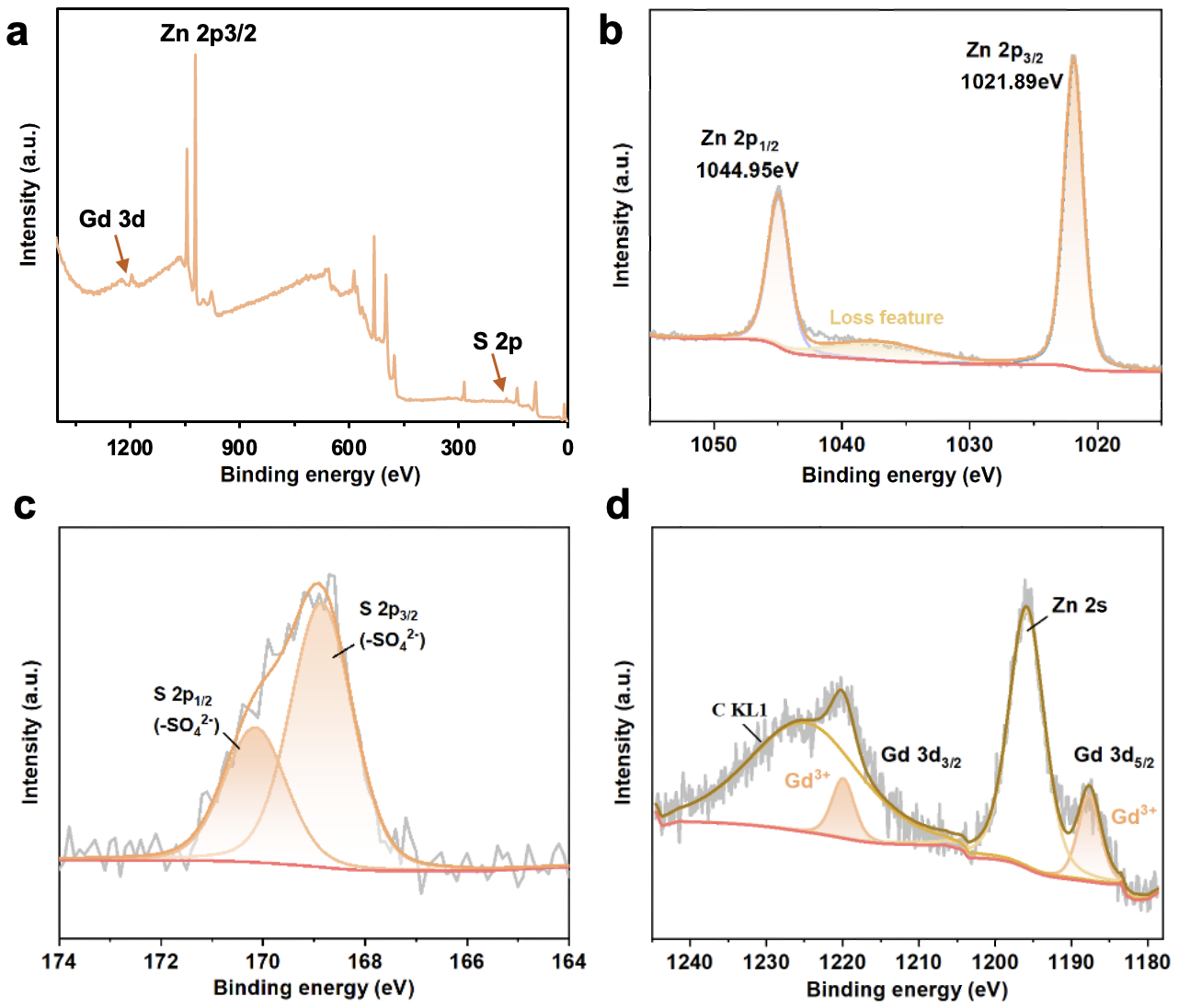


**Fig. S14** XPS spectra of the Zn anode after cycling in Zn//Zn symmetric cells for 50 h


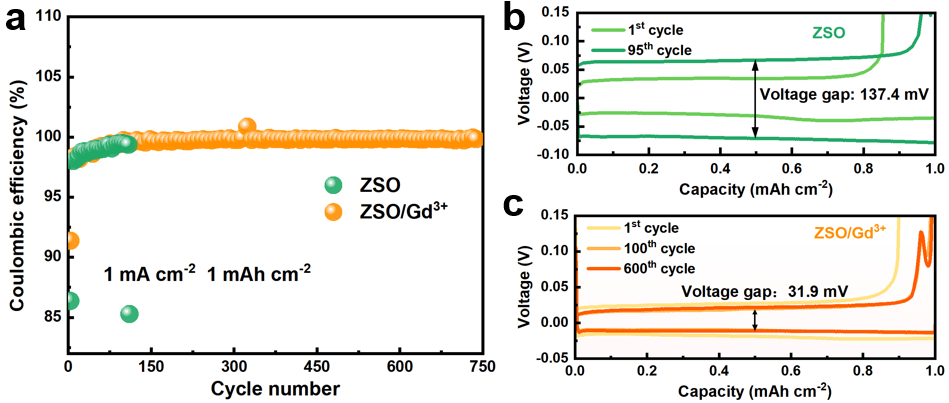


**Fig. S15** Electrochemical performance of Zn//Cu batteries. **a** Coulombic efficiency of zinc plating/stripping in Zn//Cu batteries operating at a current density of 1 mA cm^-2^ and a capacity of 1 mAh cm^-2^. Corresponding voltage-capacity curves of the Zn//Cu batteries with **b** ZSO and **c** ZSO/Gd^3+^ electrolytes


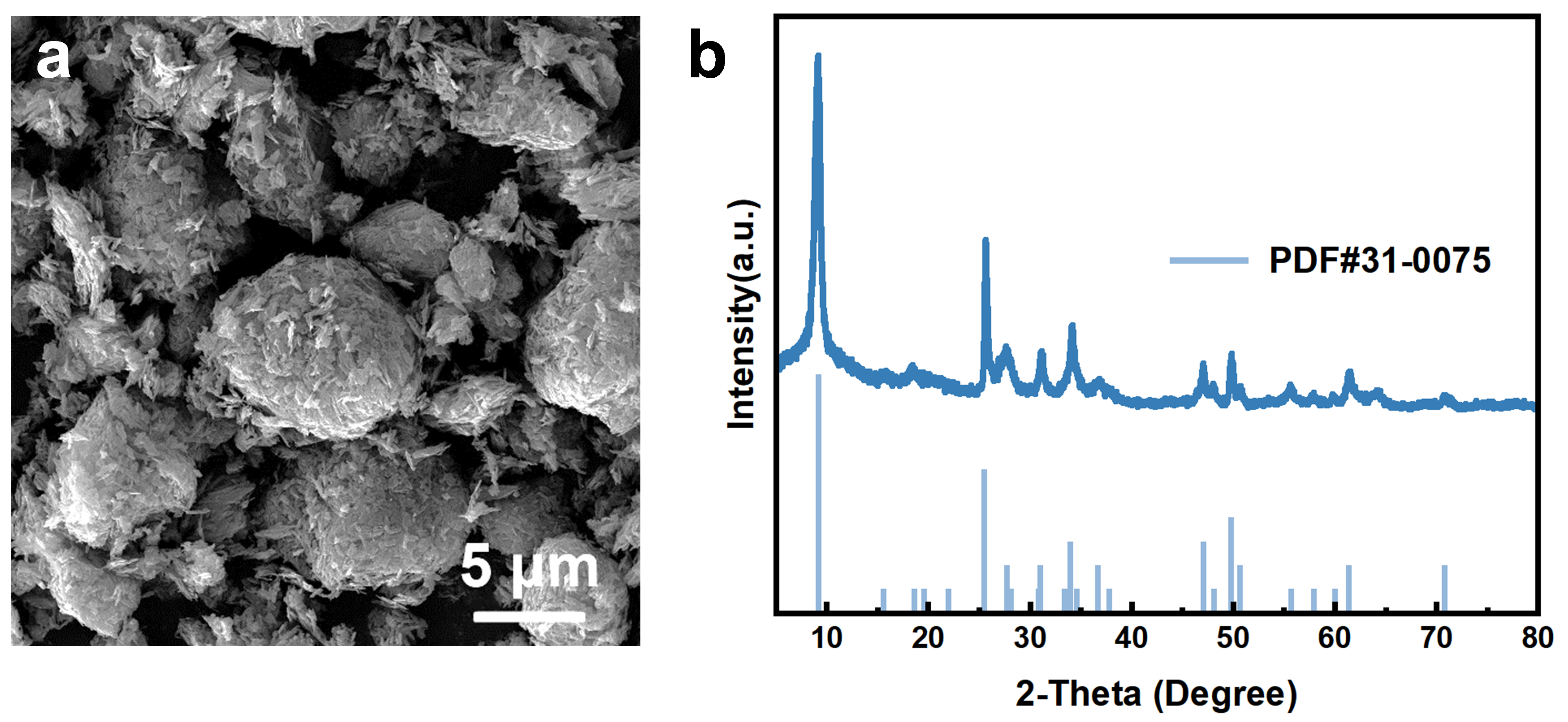


**Fig. S16** Characterization of NVO cathode material. **a** SEM image and **b** XRD pattern of NVO powders synthesized by the hydrothermal method


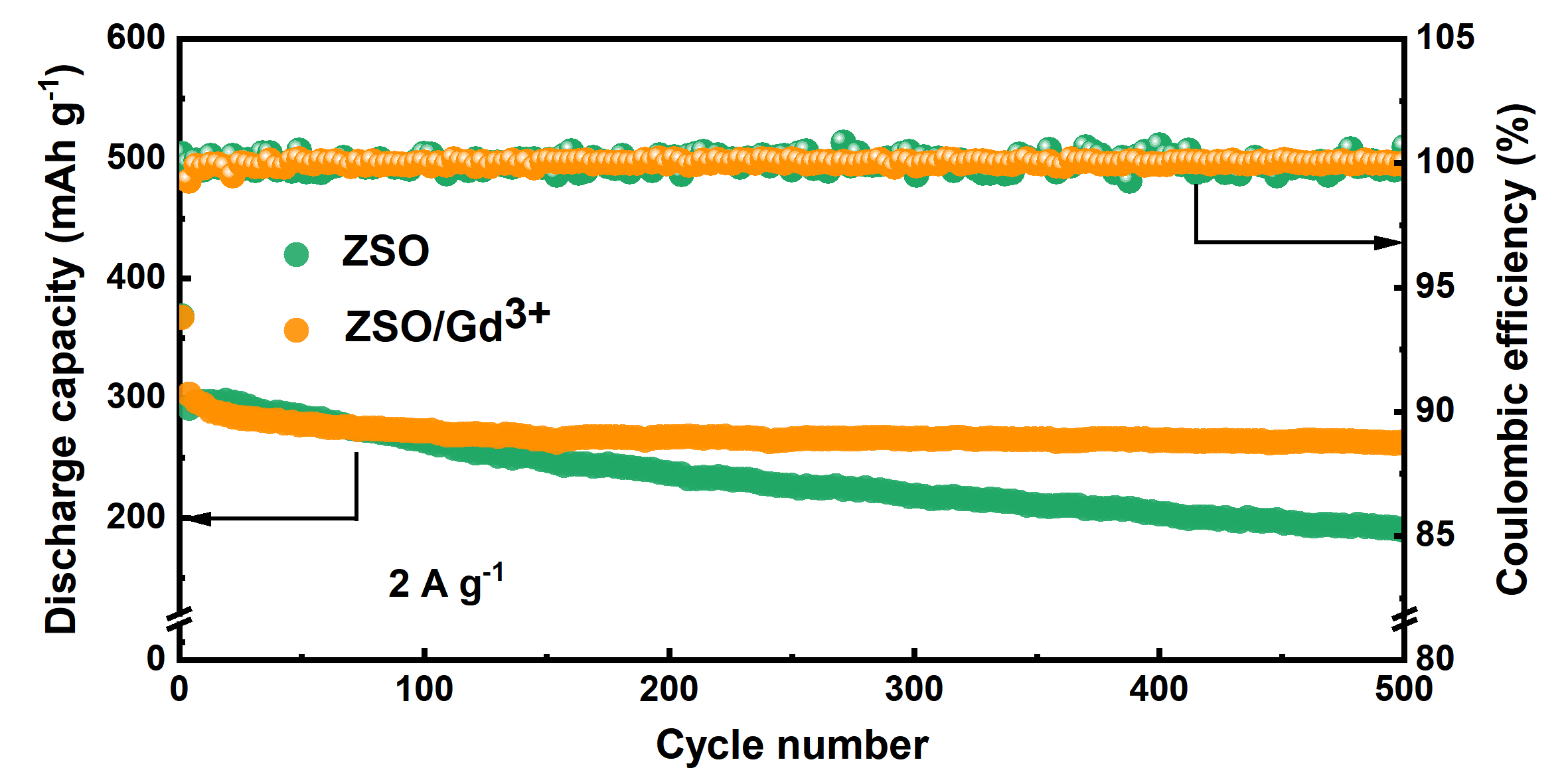


**Fig. S17** Cycling performance of Zn//NVO full cells at 2 A g^-1^


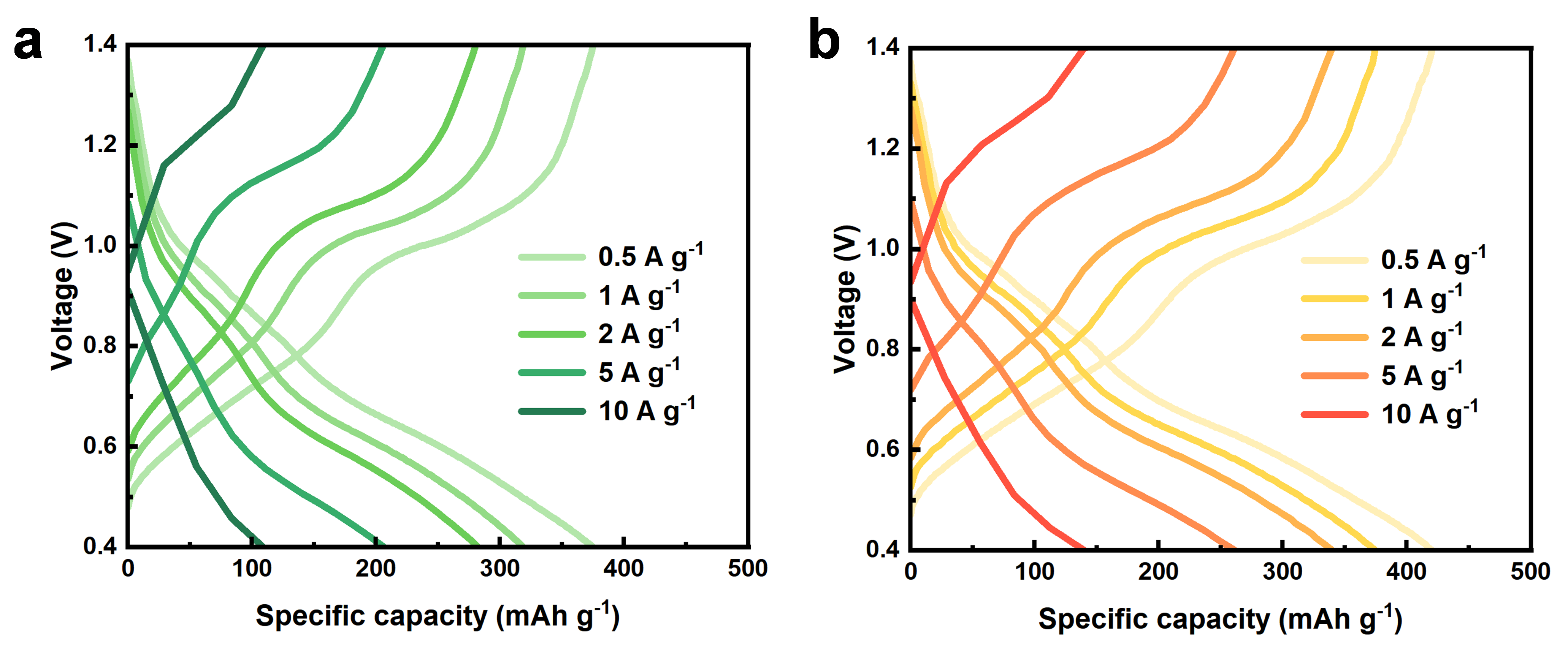


**Fig. S18** Voltage-capacity profiles of the Zn//NVO full cells with **a** ZSO and **b** ZSO/Gd^3+^ electrolytes

**Table S1** Performance comparison of AZIBs with the electrolytes containing different metallic cations

| Additive | Concentration | Symmetric cell | Full cell | Refs. |
| --- | --- | --- | --- | --- |
| Na^+^ | 2 M | NA | 82% after 1000 cycles at  4 A/g | [S1] |
| Mg^2+^ | 1 M | NA | 72.8% after 600 cycles at  2 A/g | [S2] |
| Co^2+^ | 0.2 M | NA | 92% after 5000 cycles at  4 A/g | [S3] |
| Pb^2+^ | 1.67 mM | NA | NA | [S4] |
| Li^+^ | 2 M | 100 h at 1 mA/cm^2^,  1 mAh/cm^2^ | 85.7% after 1000 cycles at 5 A/g | [S5] |
| Y^3+^ | 0.1 M | 2080 h at 5 mA/cm^2^,  2 mAh/cm^2^ | 89.6% after 2000 cycles at 5 A/g | [S6] |
| Ce^3+^ | 0.1 M | 2600 h at 2 mA/cm^2^,  1 mAh/cm^2^ | 80% after 400 cycles at 0.34 A/g | [S7] |
| Li^+^ | 1.1 M | NA | NA | [S8] |
| La^3+^ | 8.5 mM | 1200 h at 1 mA/cm^2^,  1 mAh/cm^2^ | 80% after 1000 cycles at  1 A/g | [S9] |
| Rb^+^ | 1.5 mM | 6000 h at 0.5 mA/cm^2^, 0.25 mAh/cm^2^ | 71.6% after 500 cycles at  5 A/g | [S10] |
| K^+^ | 0.2 M | 1000 h at 1 mA/cm^2^,  1 mAh/cm^2^ | 52% after 400 cycles at  0.5 A/g | [S11] |
| Ce^4+^ | 0.2 M | 2500 h at 5 mA/cm^2^,  2.5 mAh/cm^2^ | ~64% after 1000 cycles at 5 A/g | [S12] |
| Gd^3+^ | 0.05 M | 2100 h at 1 mA/cm^2^,  1 mAh/cm^2^ | 85.6% after 1000 cycles at 5 A/g | This work |

**Supplementary References**

1. F. Wan, L. Zhang, X. Dai, X. Wang, Z. Niu et al., Aqueous rechargeable zinc/sodium vanadate batteries with enhanced performance from simultaneous insertion of dual carriers. Nat. Commun. **9**, 1656 (2018). <https://doi.org/10.1038/s41467-018-04060-8>
2. Y. Zhang, H. Li, S. Huang, S. Fan, L. Sun et al., Rechargeable aqueous zinc-ion batteries in MgSO_4_/ZnSO_4_ hybrid electrolytes. Nano-Micro Lett. **12**, 60 (2020). <https://doi.org/10.1007/s40820-020-0385-7>
3. L. Ma, S. Chen, H. Li, Z. Ruan, Z. Tang et al., Initiating a mild aqueous electrolyte Co_3_O_4_/Zn battery with 2.2 V-high voltage and 5000-cycle lifespan by a Co(III) rich-electrode. Energy Environ. Sci. **11**, 2521 (2018). <https://doi.org/10.1039/c8ee01415a>
4. G. Chang, S. Liu, Y. Fu, X. Hao, W. Jin et al., Inhibition role of trace metal ion additives on zinc dendrites during plating and striping processes. Adv. Mater. Interfaces **6**, 1901358 (2019). <https://doi.org/10.1002/admi.201901358>
5. X. Guo, Z. Zhang, J. Li, N. Luo, G. Chai et al., Alleviation of dendrite formation on zinc anodes via electrolyte additives. ACS Energy Lett. **6**, 395-403 (2021). <https://doi.org/10.1021/acsenergylett.0c02371>
6. Y. Ding, X. Zhang, T. Wang, B. Lu, Z. Zeng et al., A dynamic electrostatic shielding layer toward highly reversible Zn metal anode. Energy Storage Mater. **62**, 102949 (2023). <https://doi.org/10.1016/j.ensm.2023.102949>
7. Z. Hu, F. Zhang, Y. Zhao, H. Wang, Y. Huang et al., A self-regulated electrostatic shielding layer toward dendrite-free Zn batteries. Adv. Mater. **34**, 2203104 (2022). <https://doi.org/10.1002/adma.202203104>
8. Y. Yuan, S. Pu, M. Perez-Osorio, Z. Li, S. Zhang et al., Diagnosing the electrostatic shielding mechanism for dendrite suppression in aqueous zinc batteries. Adv. Mater. **36**, 2307708 (2024). <https://doi.org/10.1002/adma.202307708>
9. R. Zhao, H. Wang, H. Du, Y. Yang, Z. Gao et al., Lanthanum nitrate as aqueous electrolyte additive for favourable zinc metal electrodeposition. Nat. Commun. **13**, 3252 (2022). <https://doi.org/10.1038/s41467-022-30939-8>
10. X. Zhang, J. Chen, H. Cao, X. Huang, Y. Liu et al., Efficient suppression of dendrites and side reactions by strong electrostatic shielding effect via the additive of Rb_2_SO_4_ for anodes in aqueous zinc-ion batteries. Small **19**, 2303906 (2023). <https://doi.org/10.1002/smll.202303906>
11. M. Xue, X. Ren, Y. Zhang, J. Liu, T. Yan. Improving aqueous zinc ion batteries with alkali metal ions. ACS Appl. Mater. Interfaces **16**, 33559-33570 (2024). <https://doi.org/10.1021/acsami.4c05372>
12. Z. Hu, F. Zhang, F. Wu, H. Wang, A. Zhou et al., Screening metal cation additives driven by differential capacitance for Zn batteries. Energy Environ. Sci. **17**, 4794-4802 (2024). <https://doi.org/10.1039/D4EE01127A>
